# Immunological Classification of Pancreatic Carcinomas to Identify Immune Index and Provide a Strategy for Patient Stratification

**DOI:** 10.3389/fimmu.2021.719105

**Published:** 2022-01-17

**Authors:** Yi Chen, Didi Chen, Qiang Wang, Yajing Xu, Xiaowei Huang, Felix Haglund, Huafang Su

**Affiliations:** ^1^ Department of Oncology-Pathology, Karolinska Institutet, Stockholm, Sweden; ^2^ Clinical Pathology and Cancer Diagnostics, Karolinska University Hospital Solna, Stockholm, Sweden; ^3^ Department of Radiation Oncology, First Affiliated Hospital of Wenzhou Medical University, Wenzhou, China; ^4^ Department of Clinical Science, Intervention and Technology (CLINTEC), Karolinska Institutet, Stockholm, Sweden

**Keywords:** pancreatic ductal adenocarcinoma (PDAC), immune characteristics, immune subtypes, immune index, immunotherapy

## Abstract

**Background:**

Cancer immunotherapy has produced significant positive clinical effects in a variety of tumor types. However, pancreatic ductal adenocarcinoma (PDAC) is widely considered to be a “cold” cancer with poor immunogenicity. Our aim is to determine the detailed immune features of PDAC to seek new treatment strategies.

**Methods:**

The immune cell abundance of PDAC patients was evaluated with the single-sample gene set enrichment analysis (ssGSEA) using 119 immune gene signatures. Based on these data, patients were classified into different immune subtypes (ISs) according to immune gene signatures. We analyzed their response patterns to immunotherapy in the datasets, then established an immune index to reflect the different degrees of immune infiltration through linear discriminant analysis (LDA). Finally, potential prognostic markers associated with the immune index were identified based on weighted correlation network analysis (WGCNA) that was functionally validated *in vitro*.

**Results:**

Three ISs were identified in PDAC, of which IS3 had the best prognosis across all three cohorts. The different expressions of immune profiles among the three ISs indicated a distinct responsiveness to immunotherapies in PDAC subtypes. By calculating the immune index, we found that the IS3 represented higher immune infiltration, while IS1 represented lower immune infiltration. Among the investigated signatures, we identified ZNF185, FANCG, and CSTF2 as risk factors associated with immune index that could potentially facilitate diagnosis and could be therapeutic target markers in PDAC patients.

**Conclusions:**

Our findings identified immunologic subtypes of PDAC with distinct prognostic implications, which allowed us to establish an immune index to represent the immune infiltration in each subtype. These results show the importance of continuing investigation of immunotherapy and will allow clinical workers to personalized treatment more effectively in PDAC patients.

## Introduction

Pancreatic ductal adenocarcinoma (PDAC) is one of the most invasive and lethal malignancies. For patients diagnosed with PDAC, the prognosis remains extremely poor, with less than a 10% survival rate (1, 2). Most patients with PDAC are diagnosed as advanced or metastatic, and the median survival time is less than 1 year. Conventional approaches using chemotherapy and radiotherapy have only moderately increased the overall survival time of patients (3). In recent years, immune checkpoint blockade (ICB) treatment based on programmed cell death protein 1 (PD-1) and its programmed death ligand 1(PD-L1) have produced promising clinical results in many types of cancers (4). However, PDAC is widely considered to be a poorly immunogenic type of cancer, and ICB treatment has thus far shown a low response rate in the treatment of PDAC. Indeed, the objective response rate in unselected PDAC patients is only 3.1% in clinical trials (5). Therefore, acquiring a complete understanding of the heterogeneity of the immune responses of patients and the mechanisms that underline the effectiveness of immunotherapy treatment for PDAC is of great importance.

In the past decade, substantial progress in research on PDAC molecular subtypes has increased our understanding of molecular pathogenesis (6, 7). Given the advancement, many attempts have been made to evaluate the expression of biomarkers to identify patients most likely to benefit from ICB. However, low patient numbers have limited observation opportunities for studying the associations between treatment response and PD-L1 expression, or microsatellite instability status. Ines de Santiago et al. performed an integrative meta-analysis of 353 patients from four different studies to derive the PDAC classification based on immunological parameters (8). The results of this study revealed that the expression characteristics related to tumor-infiltrating immune cells in different PDAC subtypes may assist in guiding immunotherapy treatments. The success of cancer immunotherapy treatment depends on multiple key steps that are involved in immune activation during the cancer immune cycle (9). The low response rates to this kind of treatment and the limited number PDAC patients who have benefitted from ICB have been attributed to low immunogenicity and multiple immunosuppressive mechanisms (10, 11). Balachandran et al. highlighted the key barriers that limit immunotherapy efficacy, including cytokines, immune cell types, and cellular components of immunosuppression (12). Thus, more detailed immune characterization of PDAC is needed in the investigation of novel therapeutic strategies.

In this study, we implemented the ssGSEA approach to evaluate immune characteristics based on the marker genes of 119 immune cells, then classified PDAC into different immune subtypes (ISs) based on the immune scores. Subsequently, the response patterns of different ISs to immunotherapy were analyzed to verify their reproducibility in different datasets. We then established the immune index to reflect the different degrees of immune infiltration in the patients. Finally, based on the co-expression network analysis, we identified potential prognostic markers related to the immune index and functionally validated them *in vitro*.

## Material and Methods

### Data Source

Gene expression profiles and clinical follow-up information data on pancreatic cancer were downloaded from the International Cancer Genome Consortium (ICGC) data portal, which contains a total of 237 samples after excluding probes with empty values and samples without clinical data. The TCGA-PAAD gene expression data (*n* = 177) and the corresponding clinical data were obtained from the UCSC Xena (http://xena.ucsc.edu/) website. An additional 250 PDAC gene expression microarray data and clinical data were derived from the NCBI Gene Expression Omnibus (GEO) database as validation cohorts, including GSE28735 (*n* = 42) (13), GSE57495 (*n* = 63) (14), GSE62452 (*n* = 66) (15), and GSE85916 (*n* = 79). Using the “Combat” algorithm in R package “sva,” the batch effects were removed, and the expression values were quantile-normalized across the different samples. The scaled estimate values derived from RNA-seq by Expectation Maximization (RSEM) were converted to transcripts per million (TPM) values by multiplying them by one million. A total of 100 immune gene signatures were obtained from the R package “IOBR” (**Table S1**) (16), and after which we calculated the immune scores using the single-sample gene set enrichment analysis (ssGSEA) algorithm in the “GSVA” package (17).

### Clustering Analysis

Based on the normalized enrichment score of the immune characteristics, the R package “ConsensusClusterPlus” (18) was used to construct a consistency matrix to classify the samples *via* clustering to generate the immune subtypes in PDAC. The “PAM” algorithm and “1-Pearson Correlation” were used to measure distance, and bootstraps were performed 500 times. Each bootstrapping process included 80% of the patients in the training set. The number of clusters was set from 2 to 10, and the optimal classification was determined through calculating the consistency matrix and the consistency cumulative distribution function.

### Immunoprofiling

To investigate the relative abundance of tumor-infiltrating immune cells, we performed CIBERSORT (19) to quantify the distribution of the 22 immune cell types in individual specimens of the ICGC cohort. Immune-related signatures of PDAC, including the Th1/IFNγ gene, cytolytic immune activity (CYT), and 47 immune checkpoints, were obtained from previous publications (20–22). Additionally, we utilized the Estimation of STromal and Immune cells in MAlignant Tumor tissues using Expression data (ESTIMATE) tool to determine the score that represents the proportion of immune and stromal cells (23). We used the “TIDE” software (24) with default parameters to analyze the differences in immune efficacy. The anti-CTLA4, anti-PD1, and anti-PD-L1 treatment profile data were obtained from previous studies (25, 26).

### LDA and Construction of the Immune Index

As different subtypes have different gene signatures, we conducted a linear discriminant analysis (LDA) for dimensionality reduction and established a subtype classification feature index to better quantify the immune characteristics of patients in different sample cohorts (27, 28). In order to do this, *z*-transformation was performed on each feature across 14 prognostic-related immune features, and the centroid of each group was dispersed as much as possible based on Fisher’s LDA optimization standards. The goal was to find one linear combination, “A,” that could maximize variance across all classes and ensure that the first two features of the model could clearly distinguish samples of the different subtypes. The LDA score was computed for the discriminative functional markers by adding the first two linear discriminants, LD1 and LD2, from the LDA of the combined normalized data to produce an immune index. The classification performance of the immune index in different subtypes was determined by the area under the curve (AUC) of multiclass receiver operating characteristic (ROC) curves.

### Weighted Correlation Network Analysis

The R software package “WGCNA” (29) was used to identify the co-expression modules in immune genes. Genes with the top 5,000 standard variations were retained and subjected to the clustering analysis in the TCGA cohort. The co-expression network conformed to the scale-free network with beta values ranging between 1 and 20. Meanwhile, the linear model was established *via* logarithms of the adjacency degree of a node (log *k*) and the appearance probability of the node [log(*p*(*k*))] with a correlation greater than 0.85. To ensure a scale-free network, the nearest soft threshold was selected and used to filter the co-expression module. In the next step, the expression matrix was converted into an adjacency matrix and then converted into a topological matrix. Based on the topological overlap matrix (TOM), we used the average-linkage hierarchical clustering method to cluster genes in order to maintain the minimum number of genes in each module of base 30 according to the standard of the hybrid dynamic cut tree. After this, we calculated the eigengenes of each module to perform a cluster analysis on the modules, then merged the modules that were closer to each other into a new module with the following characteristics: height = 0.25, DeepSplit = 2, and minModuleSize = 30. The gene networks were built and visualized by the software Cytoscape version 3.8.0 (30).

### Prognostic and Functional Enrichment Analysis

The “coxph” function of the “survival” package in R was used to perform univariate Cox analysis on significant gene modules. The prognostic performance was determined by analyzing the HR score of each gene module based on its degree of significance with *p <*0.05. Moreover, the Kyoto Encyclopedia of Genes and Genomes (KEGG) pathway and gene ontology (GO) analyses were carried out using the “clusterProfiler” package in R (31).

### Cell Culture

The human pancreatic ductal cell line PANC-1 and pancreatic carcinoma cell HPDE6-C7 were purchased from the American Type Culture Collection (ATCC, Manassas, VA, USA). The PANC-1 and HPDE6-C7 cells were maintained in DMEM medium (Gibco, Grand Island, USA) with 100 U/ml of penicillin, 100 mg/ml of streptomycin, and 10% fetal bovine serum (Gibco, Grand Island, USA) in an incubator containing 5% CO_2_ at 37°C. The cell lines were subcultured every 2 to 3 days following digestion with 1 ml 0.25% trypsin and 0.02% EDTA (Sigma-Aldrich, MO, USA). The viability was measured by the number of surviving cells as a percentage of the total number of cells. The average viability of over 95% was determined by Trypan Blue staining at 37°C in an incubator containing 5% CO_2_.

### RNA Extraction and Real-Time PCR Analysis

Total RNA from cultured cells was extracted using an RNeasy Plus Mini Kit (Qiagen, Dusseldorf, Germany). First-strand cDNA was synthesized using an M-MLV Reverse SuperScript II reverse transcription kit (Thermo Fisher Scientific, Waltham, MA, USA). The qRT-PCR reaction was performed using Power SYBR Green PCR Master Mix (Thermo Fisher Scientific, Waltham, MA, USA) on the ABI PRISM 7900 Sequence Detection System (Applied Biosystems, Foster City, CA, USA). The relative expression of genes was calculated using the 2^−ΔΔCt^ method. All experiments were performed in triplicate. The PCR primers used in this study were as follows:

hsa-CSTF2: forward, 5′-CAGCGGTGGATCGTTCTCTAC-3′ and reverse, 5′-AACAACAGGTCCAACCTCAG-3; hsa-FANGG: forward, 5′-CAGGGATTGAAGGATGTCCTCC-3 and reverse, 5′-TGGATTTCCCATCTTACGGTGA-3; hsa-ZNF185: forward, 5′-AGCTCTACCACCAAAGGGATT-3 and reverse, 5′-TGGCGAATGAGTCCTCAATGC-3; hsa-TPX2: forward, 5′-ACTTCCGCACAGATGAGCG-3 and reverse, 5′-GGATGCTTTCGTAGTTCAGATGT-3; hsa-GAPDH: forward, 5′-TGACAACTTTGGTATCGTGGAAGG-3 and reverse, 5′-AGGCAGGGATGATGTTCTGGAGAG-3.

### Small Interfering RNA and Transfection

The small interfering RNAs (siRNAs) were purchased from GEMA Gene Company (Pudong, Shanghai, China), and their sequences are listed in **Table S2**. We designed triplicate sets for each gene, and the cells were seeded into six−well plates that measured 3 × 10^5^ cells per well. The plasmids were transfected to a concentration of 2.5 µg/well using Lipofectamine^®^ 2000 reagent (Thermo Fisher Scientific, Waltham, MA, USA) according to the instructions of the manufacturer. Cells were then collected for subsequent analysis 48 h after transfection.

### Cell Viability Assay

Cell viability was determined using a Cell Counting Kit-8 (Beyotime Biotechnology, Shanghai, China). Cells were seeded into 96-well plates at a density of 2 × 103 cells per well. At 24, 48, and 72 h post-transfection, 20 μl of CCK-8 solution was added to each well. The absorbance values were recorded at a wavelength of 450 nm after a 4-h incubation period.

### Cell Invasion Assay

The cell invasion assay was performed using 8-μm Matrigel-coated Transwell inserts (Corning Costar, NY, USA) to evaluate invasion capacities *in vitro*. Dulbecco’s modified Eagle’s medium (DMEM) containing 10% FBS was added to the lower chamber. After 12 h of transfection, the cells were washed with Hanks’ balanced salt solution (Thermo Fisher Scientific, Waltham, MA, USA), suspended in 100 μl of serum-free medium (8 × 10^4^ cells), then added to the upper chamber. After 12 h of incubation, the cells were removed from the top of the filter. The cells that moved to the lower chamber were fixed with methanol and stained with 0.01% crystal violet dye, after which the number of migrated cells was counted in five random photographs under an inverted microscope (Olympus, Tokyo, Japan). The experiments were performed in triplicate.

### Statistical Analysis

The unpaired Student’s *t*-test was used for comparison between two continuous variables and a normally distributed variable. Non-normally distributed variables were analyzed using the Wilcoxon rank-sum test. To compare three or more groups, analysis of variance (ANOVA) and the Kruskal–Wallis test were performed on both the parametric method and the non-parametric method. The threshold of significance is *p*-value <0.05, <0.01, or <0.001. Different significance levels are represented in different analyses, all of which were conducted using R version 4.0.3 (R Foundation for Statistical Computing, Vienna, Austria) and GraphPad Prism version 8.0.2 (GraphPad Software, San Diego, CA, USA).

## Results

### Identification of Immune Subtypes in PDAC Based on Immune Gene Signatures

**Figure 1:**
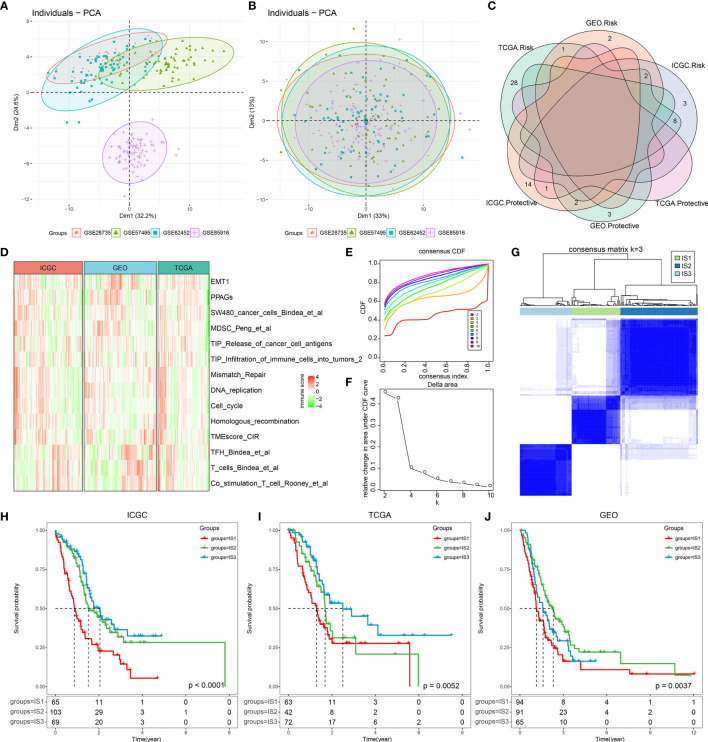
The immune-based molecular subtypes in pancreatic ductal adenocarcinoma (PDAC). **(A)** The PCA scatter plot of immune features before removing batch effects across the four involved PDAC GEO datasets. **(B)** The PCA scatter plot of immune features after removing batch effects across the four involved PDAC GEO datasets. **(C)** Overlapping of significantly prognosis-related immune features across the three cohorts (ICGC, TCGA, and GEO). **(D)** Heatmap of significantly prognosis-related immune features in at least two cohorts (*p* < 0.05). **(E)** The CDF curves for sample consensus clustering in the ICGC cohort. **(F)** Delta area curve of consensus clustering. The horizontal axis represents the category number *k*, and the vertical axis represents the relative change in area under the CDF curve. **(G)** Sample clustering heatmap when consensus *k* = 3. **(H)** Kaplan–Meier curve of the clinical outcome across the three subtypes in the ICGC cohort. **(I)** Kaplan–Meier curve of the clinical outcome across the three subtypes in the TGCA cohort. **(J)** Kaplan–Meier curve of the clinical outcome across the three subtypes in the GEO cohorts.

First, we implemented the ssGSEA approach to calculate the absolute enrichment scores of 110 immune features in the “IOBR” package from the ICGC and TCGA cohorts. Next, we used a total of 250 PDAC samples with complete follow-up and overlapping immune characteristics from GSE28735, GSE57495, GSE62452, and GSE85916 as validating cohorts and pretreated them by removing batch effects, thus leading to an independent cohort. Data before and after normalization were inspected using principal component analysis (PCA), which revealed that the batch effect was successfully removed using the “ComBat” algorithm (**Figures 1A, B**). We then performed a univariate Cox analysis, which showed that 30 immune features in the ICGC cohort, 40 immune features in the TCGA cohort, and 10 immune features in the GEO cohort were significantly associated with PDAC prognosis (**Table S3**). As shown in **Figure 1C**, there were few intersections across these three cohorts, indicating that the immune features were inconsistent among the datasets of the different platforms. Therefore, we selected 14 immune prognostic-related risk features over at least two cohorts for subsequent analysis (*p* < 0.05). The distribution and abundance of these 14 immune characteristics in the three cohorts are shown in **Figure 1D**. We then classified 237 PDAC samples, which we used as a training cohort, from the ICGC cohort according to the 14 immune features.

Using consensus clustering cumulative distribution functions (CDF) and the CDF Delta area curve, we determined that the optimal cluster number is three, at which point relatively stable clustering results can be applied and result in three PDAC ISs (**Figures 1E–G**). Upon further analysis of the prognostic characteristics of the three ISs, we observed that IS1 has the poorest prognosis, while IS3 has favorable prognosis in all three cohorts (**Figures 1H–J**), suggesting that the three immune subtypes showed consistency in different PDAC cohorts. Consequently, we compared the levels of the TNM staging system, clinical stage, and grade among the three ISs in the TCGA cohort, which was revealed to be in line with the survival data. IS3 patients account for the lowest proportion of M1 (metastasis) and the highest proportion of low-grade patients (G1, G2) (**Figure S2**).

### The Differences in the Innate and Adaptive Immune Signatures Among the Three ISs

**Figure 2:**
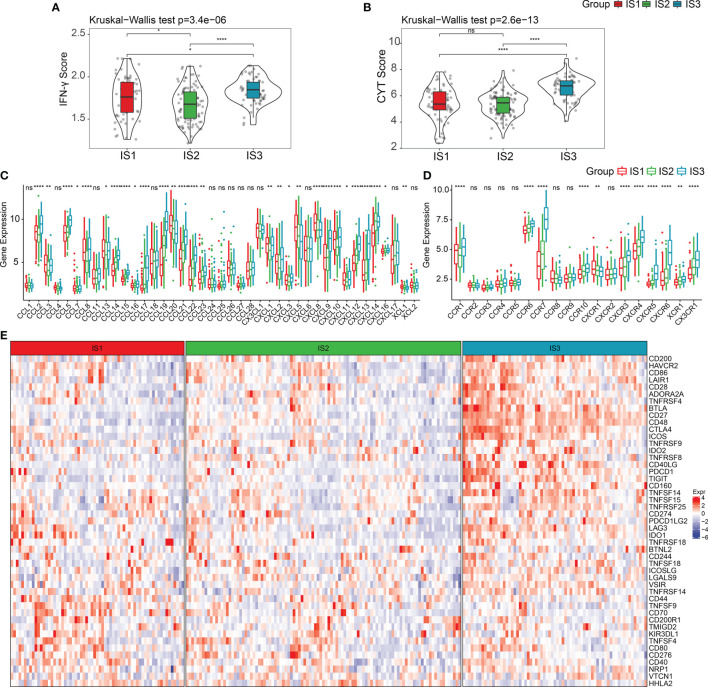
Expression of chemokines and checkpoint in PDAC immune subtypes. **(A, B)** The distribution of IFNγ scores and CYT scores in three ISs, respectively. **(C, D)** The distribution of the expression levels of chemokines and chemokine receptors across three ISs. **(E)** Expression levels of immune checkpoints in three ISs. *p < 0.05, **p < 0.01, ****p < 0.0001. ns: p > 0.05.

It is well-known that the cyclic GMP-AMP synthase-stimulator of interferon genes (cGAS-STING) signaling pathway is an innate immune pathway that can induce the release of type I IFNs and other inflammatory factors by recognizing foreign cytosolic DNAs (cDNAs) to promote innate immunity. In addition, this pathway also functions as a detector of self-DNA released from tumor cells and dying cells (32, 33). Activation of this pathway is significantly associated with tumor progression, and its role in cancer immunotherapy has been well identified in recent years (34–38). This includes pancreatic cancer, as it exhibits a strong connection to the type I interferon pathway (39–43). We compared the expression levels of four key genes in the cGAS-STING signaling pathway across the three ISs, including cGAS (encoding cGAS protein), TMEM173, TBK1, and IRF3 (encoding STING protein). As shown in **Figure S1**, there were no significant differences in the gene expressions of the four genes among the three ISs. We also found that IFNγ, a type II interferon that is predominantly produced by T helper (TH) CD4 and CD8 cytotoxic T lymphocyte (CTL) effector T cells during antigen-specific immunity, was significantly upregulated in IS3 (Kruskal–Wallis test, *p* = 3.4e−06) (**Figure 2A**). In line with these results, the cytolytic immune activity (CYT) score, which was based on the cytotoxic T-cell (CTL) markers proposed by Rooney et al. (21), consistently showed the identical trend that IS3 held the highest cytotoxic immunity (Kruskal–Wallis test, *p* = 2.6e−13) (**Figure 2B**) and was associated with positive prognosis. All together, these results suggest that our classification system accurately reflects the differences in adaptive immunity in pancreatic cancer patients rather than innate immunity.

Additionally, we observed that the expression of most chemokines and chemokine receptors in IS3 was considerably higher than in IS1 and IS2 (**Figures 2C, D**) (Kruskal–Wallis test, *p* < 0.05). Given this, we compared the expression levels of 47 immune checkpoint genes among the three ISs, and as expected, the critical inhibitory immune checkpoints [i.e., CTLA4, BTLA, IDO1, PDCD1 (PD-1), IDO2, LAG3, etc.] were overexpressed in IS3 (**Figure 2E**). Hence, we consider that targeting CTLA-4, PD-1, or PD-L1 in IS3 patients may be clinically useful.

### Difference in Immune Profiles Among the Three ISs

**Figure 3:**
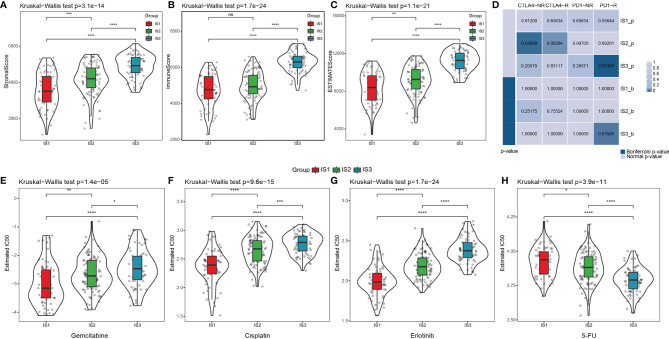
The difference of the tumor microenvironment and therapeutic treatments in PDAC immune subtypes. **(A–C)** The distribution of stromal scores, immune scores, and estimated scores in three ISs, respectively. *p < 0.05, **p < 0.01, ***p < 0.001, ****p < 0.0001. **(D)** Submap analysis of the published dataset with immunotherapy response data manifested that the IS3 could be more sensitive to the anti‐PD‐1 therapy (Bonferroni‐corrected *p* = 0.008). (Bonferroni-corrected *p* < 0.05). The distribution of the estimated IC50 for **(E)** gemcitabine, **(F)** cisplatin, **(G)** erlotinib, and **(H)** 5-FU in three ISs. *p < 0.05, **p < 0.01, ***p < 0.001, ****p < 0.0001, ns: *p* > 0.05.

In the ICGC cohort, we performed CIBERSORT analysis to evaluate the proportions of the 22 immune cells in each sample (**Table S4**). The distribution of the immune cell proportions in all samples is shown in **Figures S3A, B**, while the proportions across the three ISs are shown in **Figures S3C, D**. These results exhibit the significant differences in the immune characteristics of different subgroups. Notably, IS3 has the highest proportion of CD8^+^ T cells, resting memory CD4 T cells, and B cells, as well as the lowest proportion of M0 and M2 macrophages. Immune infiltration analysis showed that IS3 has the highest immune infiltration, followed by IS2, while IS1 had the lowest stromal and immune score (**Figures 3A–C**; **Table S5**). All together, we observed that IS3 patients presented a preferable cell-mediated immune response and humoral immune response.

Subsequently, we implemented the TIDE algorithm to evaluate the potential clinical effects of immunotherapy in the three ISs we defined. As shown in **Figure S4A**, there was apparently no difference in the TIDE scores of the three ISs. At the same time, IS3 had the highest T-cell dysfunction score compared with IS1 (*p* < 0.0001) and IS2 (*p* < 0.0001), but the lowest score in T-cell exclusion (*p* < 0.0001) (**Figures S4B, C**; **Table S6**). These results may partially explain why T-cell infiltration was the highest in the IS3 patients, but the objective response rates of ICB in clinical trials were extremely low. Therefore, there are likely other underlying mechanisms that lead to T-cell dysfunction due to sustained antigen exposure. We used submap analysis to compare the similarity between the gene signatures of the three ISs and melanoma patients treated with anti-PD1 and anti-CTLA4 (44), which revealed that IS3 patients were more sensitive to PD-1 inhibitors than the other two subtypes (**Figure 3D**). We also analyzed the responsiveness of traditional chemotherapy drugs, including gemcitabine, cisplatin, erlotinib, and fluorouracil (5-FU), in all three ISs. IS1 was more sensitive to gemcitabine (*p* < 0.01 and *p* < 0.0001, respectively), cisplatin (*p* < 0.0001), and erlotinib (*p* < 0.0001) compared with IS2 and IS3, while IS3 was more sensitive to 5-FU (*p* < 0.0001) (**Figures 3E–H**). Thus, our overall computational calculations predicated that IS3 has the highest immune cell infiltration. Therefore, stratification might be helpful in patient selection for ICB trials or further studies on drug resistance, regardless of the wide ineffectiveness with PDAC treatment so far.

### Construction of the Immune Index and Its Related Immune Characteristics

**Figure 4:**
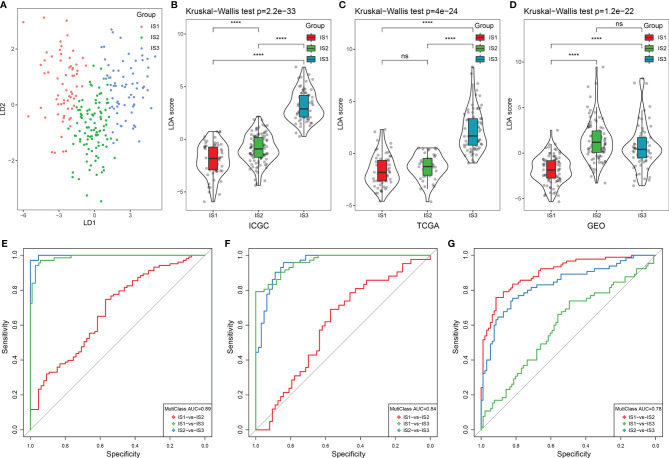
LDA score and immune index construction. **(A)** The relationship between the first two linear discriminants and three ISs. **(B–D)** The distribution of the LDA scores across three ISs in the ICGC, TCGA, and GEO cohorts. **(E–G)** The ROC curve of the immune index in the ICGC, TCGA, and GEO cohorts. ****p < 0.0001. ns: *p* > 0.05.

To further assess the differences in the gene signatures across the three ISs, as well as quantify the immune characteristics of individual patients, we performed LDA analysis. As shown in **Figure 4A**, the first linear discriminants (LD) of the model clearly distinguish between the samples of different subtypes. Following this, we calculated the LDA score of each patient in the three cohorts, which make up the immune index. The differences in the scores were significant across the three ISs (**Table S7**; **Figures 4B–D**). The classification performance of the immune index in the different subtypes is depicted in **Figure 4E** with a multiclass comprehensive prediction AUC of 0.89. After applying the immune index to the TCGA and GEO cohorts, we observed a similar performance to the ICGC dataset (multiclass AUC = 0.84 and 0.78, respectively) (**Figures 4F, G**). Together, a high index indicated a higher immune infiltration as seen in IS3 and vice versa as seen in IS1.

Additionally, to observe the relationship between the immune index and immune cell characteristics, the immune scores in 28 immune cells were calculated using ssGSEA analysis (**Table S8**) (45). Most of the immune cells in IS3 had significantly higher enrichment scores than IS1, including activated B cells, activated CD8 T cells, activated CD4 T cells, T follicular helper cells, myeloid-derived suppressor cells (MDSCs), and natural killer cells, all of which were significantly positively correlated with the highest immune index (**Figures S5A–C**).

**Figure 5:**
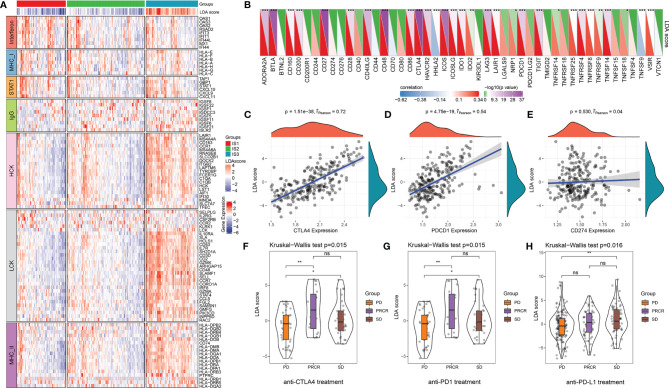
The relationship between the immune index and inflammatory characteristics and immune checkpoints. **(A)** The abundance of seven inflammatory metagenes and immune index in three ISs. **(B)** The correlation between the immune index and expression levels of immune checkpoints. *p < 0.05, **p < 0.01, ***p < 0.001, Spearman rank correlation. In the upper triangle, the shade of color represents the level of −log10(*p*-value), while in the lower triangle, the shade of color represents the level of correlation. **(C–E)** The correlation between the immune index and expression levels of CTLA4, PDCD1, and CD274 (Spearman rank correlation). **(F)** The distribution of the immune index in different treatment response statuses after anti-CTLA4 treatment. **(G)** The distribution of immune index in different treatment response statuses after anti-PD1 treatment. **(H)** The distribution of the immune index in different treatment response statuses after anti-PD-L1 treatment. *p < 0.05, **p < 0.01, ns: *p* > 0.05. PD, progressive disease; PRCR, partial response and complete response; SD, stable disease.

Recent studies have revealed that the recruitment of tumor-associated immune cells that occurs during inflammation that accompanies tumor progression has been shown to promote tumor growth and contribute to angiogenesis, invasion, and metastasis (46–48). Therefore, we also analyzed the distributions of inflammation-related genes of the ISs in the ICGC cohort, the results of which revealed that the monocyte/myeloid lineage (HCK), T cells (LCK), major histocompatibility class II complex (MHC-II) molecules, and gene clusters are all highly expressed in IS3 compared with IS1 and IS2 (**Figure 5A**; **Table S9**). All seven inflammation-related metagenes were then quantified with the enrichment score using the ssGSEA approach as shown in **Figure S6A**, which revealed that the enrichment score of the five metagenes in IS3 are significantly higher than in IS1 and IS2 (*p* < 0.0001), with the exception of MHC-I and interferon. The relationship between the LDA score and the enrichment score was identified using Pearson’s correlation coefficient. Based on this, we found that antitumor immune metagenes, including HCK, LCK, MHC-II, and STAT1 were significantly positively correlated with the immune index (*p* < 0.001) (**Figure S5B**).

The correlation between the immune index and the expression levels of 47 immune checkpoints is illustrated in **Figure 5B**. Notably, the LDA score has a significant positive correlation with many critical inhibitory immune checkpoints [i.e., CTLA4, BTLA, IDO1, PDCD1 (PD-1), IDO2, LAG3, etc.]. Of those, CTLA4 (*r* = 0.72, *p* < 0.0001) and PDCD1 (*r* = 0.54, *p* < 0.0001) show high correlation, whereas no significant correlation between the immune index and CD274 was discovered (*r* = 0.04, *p* = 0.530) (**Figures 5B–D**). We then analyzed the difference between immune index and melanoma patients treated with anti-PD1 and anti-CTLA4 as well as urothelial carcinoma patients treated with PD-L1 under different responses. The LDA score was significantly upregulated in patients with an immunotherapy response compared with SD or PD patients in anti-CTLA4 and anti-PD1 treatments (**Figures 5F, G**). However, there was no big difference in anti-PD-L1 patients across the three ISs (**Figure 5H**). These results reinforce the results of the submap analysis in **Figure 3D** that indicate that immune index can be a good representation of the gene profile. It also further suggests that patients with high immune infiltration (IS3) might benefit from anti-PD-1 therapy.

### Identification of the Co-Expression Gene Module of the Immune Index

**Figure 6:**
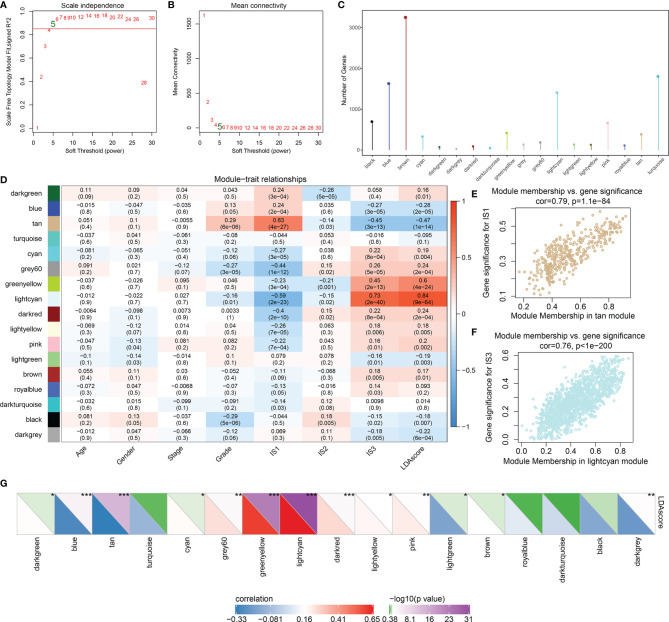
Identification of the co-expressed gene modules of the immune index. **(A)** Analysis of the scale-free fit index for various soft-thresholding powers (*β*). **(B)** Analysis of the mean connectivity for various soft-thresholding powers. **(C)** The distribution of gene numbers in each module. **(D)** The relationships between gene modules and clinical variables, ISs, and immune index. **(E)** Scatter plot of module members versus gene significance for IS1 in the tan module. **(F)** Scatter plot of module members versus gene significance for IS1 in the light cyan module. **(G)** The correlation between immune index and gene modules. In the upper triangle, the shade of color represents the level of −log10(*p*-value), while in the lower triangle, the shade of color represents the level of correlation. *p < 0.05, **p < 0.01, ***p < 0.001.

According to the weighted correlation network analysis (WGCNA) method described previously, samples were clustered (**Figure S6A**) and immune gene co-expression modules were identified with a soft threshold of 5 for the scale-free network (**Figures 6A, B**). As a result, a total of 18 gene modules were generated based on a dissimilarity measure (1-TOM), where the gray module was a collection of genes that could not be gathered into other modules (**Figures 6C**, **S6B**; **Table S10**). Furthermore, we analyzed the correlation between each module and the age, gender, clinical stage, grade, IS1, IS2, IS3, and LDA score of the patient. As shown in **Figure 6D**, the tan module showed a significant positive correlation with IS1 and a negative correlation with IS3 and its LDA score. On the other hand, the light cyan module showed the opposite result. The genes in these two modules are significantly associated with gene significance and module membership (IS1 is tan and IS3 is light cyan) (**Figures 6E, F**).

**Figure 7:**
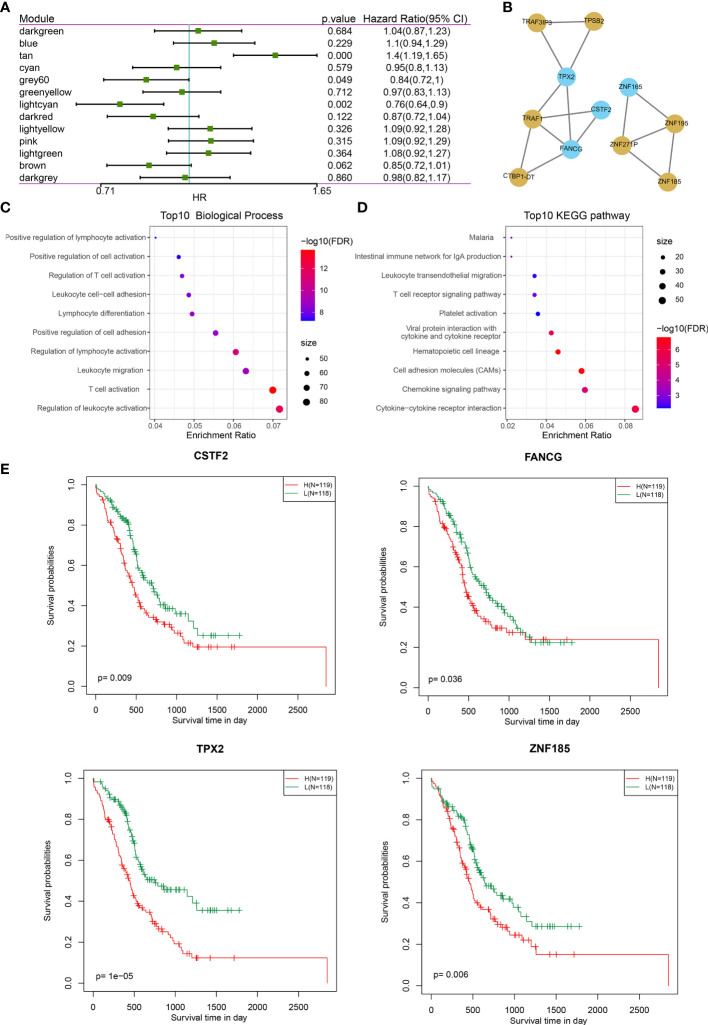
Co-expression gene module features and the prognostic association. **(A)** Forest plot of the univariate analyses of gene modules with overall survival. **(B)** Co-expression network of 11 potential gene markers related to immune index. Blue color indicates genes derived from the light cyan module; yellow color indicates genes derived from tan module. **(C, D)** The functional enrichment of the module tan: **(C)** top 10 GO biological process; **(D)** top 10 KEGG pathways. **(E)** The gene expression levels of four genes (CSTF2, TPX2, FANGG, and ZNF185) were significantly associated with overall survival (log-rank test, *p* < 0.05).

Next, we investigated the correlation between each of these gene modules and the immune index. In this analysis, each of these 13 modules were significantly associated with the LDA score (**Figure 6G**) where tan is shown as a risk factor (*p* < 0.0001, HR = 1.4) and light cyan is shown as a favorable factor (*p* = 0.002, HR = 0.76) (**Figure 7A**; **Table S11**), as expected. We subsequently screened the co-expressed genes of prognostic-related modules with co-expression weights greater than 0.35 and identified 11 hub genes (**Figure 7B**; **Table S12**). These hub genes reflected the immune index and showed up as potential candidates for biomarkers. Functional enrichment analysis revealed that genes within tan modules were enriched for biological terms (GO) or KEGG pathways with strong significance in homologous recombination, the p53 signaling pathway, DNA replication, and the cell cycle (**Figure S7**). Meanwhile, the light cyan module was significantly associated with immune-related processes including positive regulation of lymphocyte activation, positive regulation of cell activation, regulation of T-cell activation, etc. (**Figures 7C, D** **)**. Among these hub genes, high expressions of CSTF2, TPX2, FANCG, and ZNF185 were significantly associated with poor outcomes (log rank test, *p* = 0.009, *p* < 0.0001, *p* = 0.0357, and *p* = 0.006, respectively) (**Figures 7E** and **S8**).

These data suggest that the expression levels of CSTF2, TPX2, FANGG, and ZNF185 may be negatively correlated with the immune index and warrant validation of their biological functions *in vitro* to find potential biomarkers indicative of the immune index.

**Figure 8:**
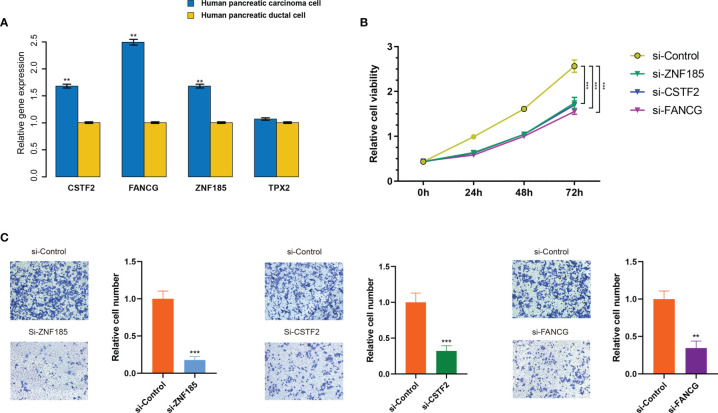
ZNF185, CSTF2, and FANCG were overexpressed in PDAC cell lines and promote cell viability and invasion. **(A)** Expression levels of ZNF185, CSTF2, FANCG, and TPX2 in human pancreatic ductal cell line PANC-1 and pancreatic carcinoma cell HPDE6-C7. Data are presented as mean ± standard error based on at least three independent experiments. **p < 0.01, ***p < 0.001. **(B)** CCK-8 assay on HPDE6-C7 cell line co-transfected with si-control and the optimal si-ZNF185, si-CSTF2, and si-FANCG. **(C)** The invasion assays on HPDE6-C7 cell line co-transfected with si-control and the optimal si-ZNF185, si-CSTF2, and FANCG.

Given that in biological and therapeutic systems upregulated genes are more likely to be manipulated than downregulated genes, we decided to focus our validation study on investigating the functions of CSTF2, TPX2, FANCG, and ZNF185 in PDAC. Firstly, we examined the expression levels of these genes in the human pancreatic ductal cell line PANC-1 as well as the pancreatic carcinoma cell HPDE6-C7. As presented in **Figure 8A**, increased expression of ZNF185, CSTF2, and FANCG (*p* < 0.01), but not TPX2, was observed in the HPDE6-C7 cell line (*p* > 0.05). These results indicate that ZNF185, CSTF2, and FANCG were overexpressed in PDAC and might be considered as biomarkers of poor prognosis.

Next, for each gene (ZNF185, CSTF2, and FANCG), we designed triplicate sets of siRNAs. We downregulated ZNF185, CSTF2, and FANCG in HPDE6-C7 through transfection with three siRNAs using the empty vector as a negative control. As a result, gene expressions significantly dropped after transfection (**Figure S10**), where si-ZNF185-3, siCSTF2-2, and si-FANCG-3 exhibited the best transfection efficiency among each group (*p* < 0.01, *p* < 0.001, and *p* < 0.001, respectively). These siRNAs were subsequently selected for functional cell experiments. Weakened cell viabilities were observed in ZNF185, CSTF2, and FANCG downregulated HPDE6-C7 cells compared with NC-transfected cells after 24, 48, and 72 h (*p* < 0.001; **Figure 8B**). Additionally, the migration assay showed that downregulation of ZNF185, CSTF2, and FANCG in HPDE6-C7 cells can significantly inhibit cell invasion capability (*p* < 0.01; **Figure 8C**). Collectively, these findings indicate that ZNF185, CSTF2, and FANCG are involved in the proliferation and metastasis of PDAC.

## Discussion

During the last decade, the emergence of immunotherapy has presented a significant advantage for certain malignancies, including melanoma, lung cancer, and other tumor patients (49–52). This has resulted in the improvement of the prognosis of treated patients, suggesting that there could be substantial benefits associated with immunotherapy. Even though immunotherapy has achieved impressive clinical benefits, its applications have shown limited efficacy due to multiple immune escape mechanisms. There is an urgent need to better understand the tumor immune microenvironment to design more effective strategies and improve the response and outcomes of immunotherapy. In this study, we reevaluated available OMICS data from an immunological perspective to understand the immune response of PDAC and provide clinical implications for personalized immunotherapy.

Pancreatic tumors avoid immune responses through different mechanisms. First, pancreatic TME is rich in immunosuppressive cells such as MDSCs, regulatory T cells (Tregs), tumor-associated macrophages (TAMs), and immunosuppressive antigen-presenting cells (APCs), which lead to refractory ICB (12, 53, 54). In our study, unsupervised consensus clustering showed that there is substantial variation in the immune characteristics of PDAC. Among all subtypes in our study, IS3 is associated with the best prognosis, whereas IS1 indicates the poorest survival probability. Cyclic GMP-AMP synthase (cGAS) is a nucleotidyl transferase that is critical for the recognition of double-stranded DNA in the cytosol and generates second messenger cyclic GMP-AMP (cGAMP). It promotes the activation of STING to elicit kinase TBK1 and its downstream substrate, the transcription factor IRF3. The phosphorylated IRF3 then translocates into the nucleus and stimulates the production of type I interferons (IFNs) and other cytokines, leading to immunity regulation (55, 56). Type I IFNs are antitumor cytokines and modulators of innate immune activation that have recently been shown to cause radiosensitization in pancreatic cancer, and they all bind to the cell surface receptor complex (IFNAR) (39, 43, 57). The deactivation of the IFN1–IFNAR1 pathway by cancer-associated fibroblasts (CAFs) leads to tumorigenic effects and tumor growth in pancreatic cancer (41, 43). This pathway affects the pancreas by regulating the production of T cells so that when STING is activated in human cells, the T-cell infiltration can be decreased (41). However, our findings have not shown a significant difference in gene expressions of these four key genes within cGAS-STING pathways among the three ISs, suggesting that our classifications have little connection with innate immunity in pancreatic cancer. Furthermore, it is well known that IFN-γ has been shown to exhibit antitumor activity both *in vitro* and *in vivo*, and deficiencies in IFN-γ (IFN-γ^−/−^) or the IFN-γ receptor (IFN-γR^−/−^) promote tumor development in mice (58, 59). The CYT score has been reported to be associated with improved prognosis in glioma, gastric cancer, and hepatocellular carcinoma, among other cancers (21, 60–62). In our study, IS3 patients represent the highest score in both IFN-γ and CTY. Their maintenance of strong immune activity may suggest a good response to immunotherapy. Since most of the expression levels of inhibitory immune checkpoints were upregulated more in IS3 than in other ISs [i.e., CTLA4, BTLA, IDO1, PDCD1 (PD-1), IDO2, LAG3, etc.], we can conclude that different subgroups have varied therapeutic responses to ICB, which indicates that IS3 may be sensitive to ICB and vice versa.

We also demonstrated stromal and immune phenotypes in IS3, including a high proportion of T cells CD8, T cells CD4 memory resting, and B cells as well as a low proportion of macrophages M0 and macrophages M2 based on CIBERSORT and ESTIMATE algorithms. The presence of M2-type macrophages is associated with poor clinical outcomes in various types of cancers due to certain cellular interactions within metastatic sites. Nevertheless, the low CD8 T cells and the high M2-type macrophages were infiltrated in IS1, with unsurprisingly minimal immune infiltration. Our study revealed that IS3 is more significantly correlated with melanoma patients who respond well to anti-PD1 treatment than IS1 and IS2. However, the high levels of T-cell dysfunction in IS3 might explain why some PDAC patients have high T-cell infiltration but are generally tolerant to ICB. Additionally, IS1 patients were more sensitive to traditional chemotherapeutic agents, including gemcitabine, cisplatin, and erlotinib, but more chemoresistant to 5-FU. These findings might provide a strong rationale in patient selection for systemic therapy option-combination regimens.

Unlike PCA, which summarizes the total variation in a dataset, LDA derives synthetic variables from a linear combination of features looking for maximum separation of two or more classes of objects. This allows us to derive an immune index to characterize immunity based on LDA scores. Consistent with the highest immune infiltrations, IS3 represented the highest immune index, which showed as significantly positively associated with the immune cells engaged in the antitumor response. High LCK has been identified with a good prognosis in breast cancer, endometrial carcinoma, and melanoma (63–65). It was in this way that we first identified the potential roles of LCK in PDAC. Strikingly, Tiziana et al. reported that MHC-II was highly correlated with the LCK and HCK metagene (66), which reflects the infiltration of T cells and monocytes (67). Indeed, IS3 shows high immune infiltration and plays critical roles in antitumor immune responses through activating MHC-II, LCK, and HCK as well as augmentation of its expression levels. Pancreatic tumors remain refractory to ICB as it is characterized by its T-cell exclusion and an immunosuppressive tumor microenvironment (68–70). However, while the LDA score is significantly associated with the expression levels of anti-CTLA4 and anti-PD1, it is not associated with PD-L1. The high immune index represents a good clinically predicted response to both anti-CTLA4 and anti-PD1 therapy. Actually, our results strengthen the findings that patients with a high immune index (IS3) and low T-cell exclusion might benefit from PD-1 therapy; however, the complications resulting from severe T-cell dysfunction needs to be further investigated in the future.

We found several interesting components within the hub gene modules related to IS3 as well overall survival: ZNF185, FANCG, and CSTF2. These candidates have been functionally validated, and their potential roles have also been identified by previous studies. ZNF185, an actin cytoskeleton-associated protein from the LIM family of Zn-finger proteins, has been demonstrated to be a bona fide p53 target gene following DNA damage, which is consistent with the enrichment results of the GO biological process in the tan module–p53 single signaling pathway (71). In addition, the overexpression of ZNF185 has been shown to promote chemoresistance, tumor proliferation, and inhibition of apoptosis by downregulating SMAD4 in PDAC (72). Furthermore, inherited mutations of the DNA repair genes FANCG have been thoroughly demonstrated to be responsible for accelerating genomic instability due to the loss of the G1/S checkpoint, leading to increased risk in PDAC (73, 74). Even though the functional role of CSTF2 has not been conclusively proven, the overexpression results in the shortening of 3′ UTRs and promotes pathogenesis and poor prognosis in several types of cancer, including endometrial carcinoma, lung cancer, and bladder cancer (75–77). The results indicate that ZNF185, FANCG, and CSTF2 cannot be effective biomarkers for risk assessments and reflect the immune index for personalized treatment decision-making in PDAC. Nevertheless, the immune-related roles of these genes will have to be further verified in future studies.

Although computational approaches for analyzing bulk RNA sequencing data from public databases have been well established, this study shares some limitations with previous bioinformatics studies. Given the recalcitrant nature of pancreatic cancer, there are rarely research cohorts regarding immunotherapy for it. The TCGA, ICGC, and GEO cohorts were collected prior to the immunotherapy. The projection of pancreatic cancer by assessing the similarities between molecular features in gene expression data with melanoma immunotherapy cohorts may cause some bias. Furthermore, given that we used these three gene signatures to represent the immune infiltration, we might be unable to comprehensively profile the immune landscape of the PDAC sample. The true predictive effect of these three gene signatures (ZNF185, FANCG, CSTF2) on immune infiltration needs to be evaluated in future studies. Although we predict that the IS3 group may benefit from anti-PD1 therapy and show low levels of T-cell exclusion, whether the true response rate is higher than in other groups requires further validation in other cohorts receiving anti-PD1 treatment.

In summary, we identified three immunological subtypes of PDAC, each with distinct immune infiltration and prognostic characteristics. We further established an immune index to quantify the abundance of immune infiltration in different ISs. Finally, based on the co-expression network analysis, potential biomarkers (ZNF185, FANCG, CSTF2) related to the immune index were identified and functionally validated *in vitro*. Overall, our results provide new insights into the stratification and selection of patients for personalized immunotherapy assessment in PDAC.

## Data Availability Statement

The original contributions presented in the study are included in the article/**Supplementary Material**. Further inquiries can be directed to the corresponding author.

## Author Contributions

Conceptualization: YC, DC, and HS. Methodology: YC, DC, and HS. Software: YC, QW, YX, and XH. Validation: YC, YX, XH, and HS. Formal analysis: YC, DC, QW, YX, XH, and HS. Data curation: all authors. Writing—original draft preparation: YC, DC, and HS. Writing—review and editing: YC, DC, FH, and HS. Visualization: YC and QW. Supervision: HS. Project administration: HS. Funding acquisition: HS. All authors contributed to the article and approved the submitted version.

## Funding

This work was supported by the ZheJiang Province Public Welfare Technology Application Research Project (number 2021KY782) and the Wenzhou Municipal Science and Technology Bureau (number Y2020151).

## Conflict of Interest

The authors declare that the research was conducted in the absence of any commercial or financial relationships that could be construed as a potential conflict of interest.

## Publisher’s Note

All claims expressed in this article are solely those of the authors and do not necessarily represent those of their affiliated organizations, or those of the publisher, the editors and the reviewers. Any product that may be evaluated in this article, or claim that may be made by its manufacturer, is not guaranteed or endorsed by the publisher.

## Supplementary Material

Supplementary Figure 1 The distribution of the expression levels of four key genes in the innate immune regulatory cGAS-STING signaling pathway across three ISs.

Supplementary Figure 2 The fraction of Stage T, Stage N, Stage M, clinical stage, and grade in three ISs.

Supplementary Figure 3 The difference of immune cells in three ISs **(A)**. The fraction of 22 immune cells in three ISs. **(B)** The distribution of the estimated immune cell proportions in all samples. **(C)** The distribution of the estimated immune cell proportions in three ISs. **(D)** The abundance of immune cell components in three ISs, only the immune cells in which the nonzero proportions in all samples exceeded half were retained

Supplementary Figure 4
**(A–C)** The differences of TIDE scores, T cell dysfunction scores, and T cell exclusion scores in three ISs, respectively.

Supplementary Figure 5 The relationship between the immune index and immune-related characteristics. **(A)** The abundance of 28 immune cell scores in three ISs. **(B)** The distributions of 28 immune cell scores in three ISs. **p* < 0.05; ***p* < 0.01, ****p* < 0.001. **(C)** The correlations between immune index and immune cell scores. In the upper right triangle, the shade of color represents the level of correlation, while in the lower left triangle, the shade of color represents−log10(p value). **p* < 0.05; ***p* < 0.01, ****p* < 0.001.

Supplementary Figure 6 T **(A)** The distribution of seven inflammatory metagenes in three ISs. **p* < 0.05; ***p* < 0.01, ****p* < 0.001, *****p* < 0.0001. **(B)** The correlations between the LDA score and inflammatory metagene enrichment score. In the upper right triangle, the shade of color represents the level of correlation, while in the lower left triangle, the shade of color represents−log10 (p value). **p* < 0.05; ***p* < 0.01, ****p* < 0.001.

Supplementary Figure 7
**(A)** WGCNA sample clustering. **(B)** Dendrogram of all differentially expressed genes clustered based on a dissimilarity measure (1-TOM).

Supplementary Figure 8 The functional enrichment of module lightcyan, **(A)**, top 10 GO biological process; **(B)**, top 10 KEGG pathways

Supplementary Figure 9 Kaplan-Meier curves show the overall survivals between high- and low- expression of seven hub genes with no significance (CTBP1-DT, ZNF165, TPSB2, ZNF195, TRAF1, TRAF3IP3, ZNF271P).

Supplementary Figure 10 The relative expression of ZNF185, CSTF2, and FANCG after transfection of relevant siRNAs in PANC-1 cell line. Error bars represent the standard error of the mean from at least three independent experiments). **p* < 0.05; ***p* < 0.01, ****p* < 0.001.
