# Supplementary figures and images for: Immunological Classification of Pancreatic Carcinomas to Identify Immune Index and Provide a Strategy for Patient Stratification
Source: Front Immunol. 2022 Jan 17;12:719105. doi: 10.3389/fimmu.2021.719105 (PMC8801451; doi:10.3389/fimmu.2021.719105)

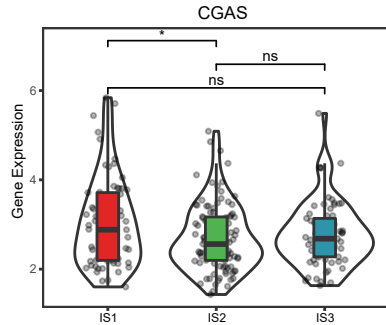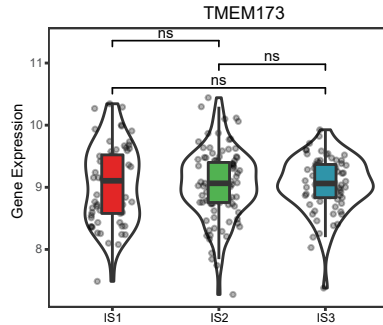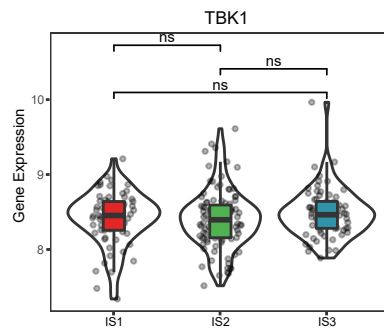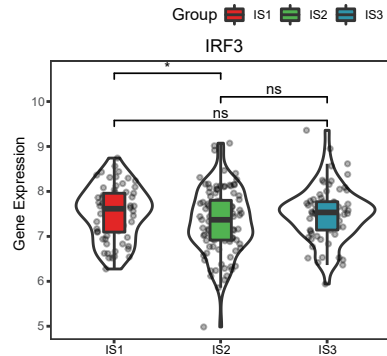

Supplement: Supplementary Figure 1 — The distribution of the expression levels of four key genes in the innate immune regulatory cGAS-STING signaling pathway across three ISs. [file DataSheet_1.zip › Supplementary materials/FigS1.pdf]

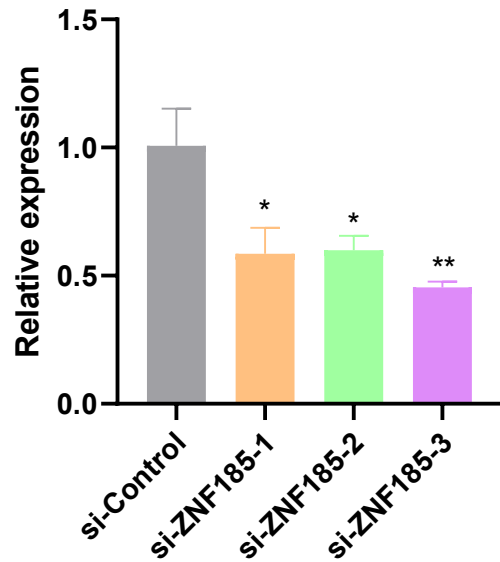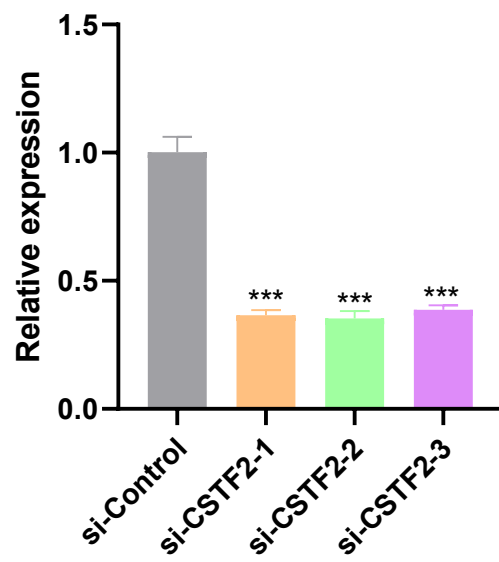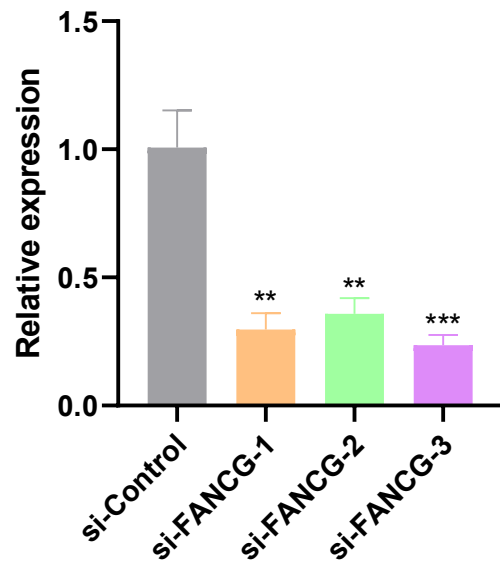

Supplement: Supplementary Figure 1 — The distribution of the expression levels of four key genes in the innate immune regulatory cGAS-STING signaling pathway across three ISs. [file DataSheet_1.zip › Supplementary materials/FigS10.pdf]

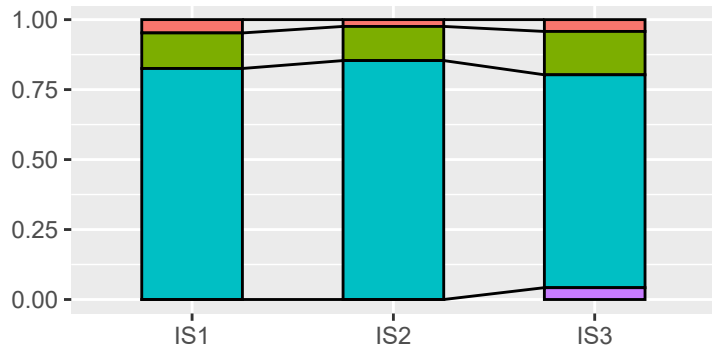

Stage T T1 T2 T3 T4

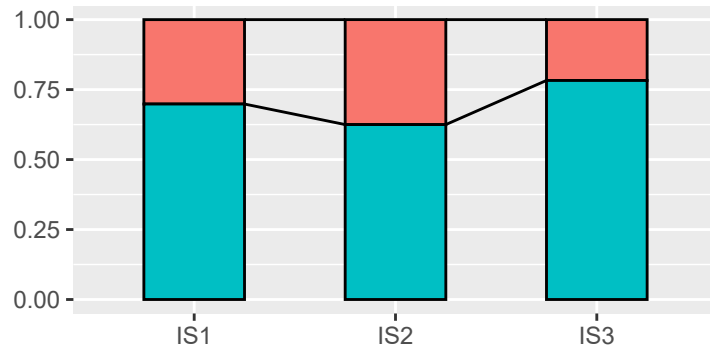

Stage N N0 N1

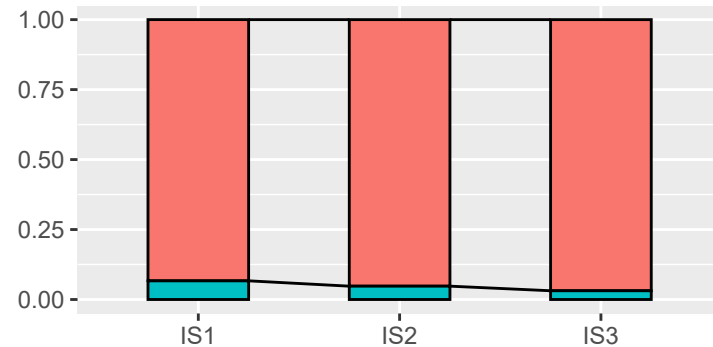

Stage M M0 M1

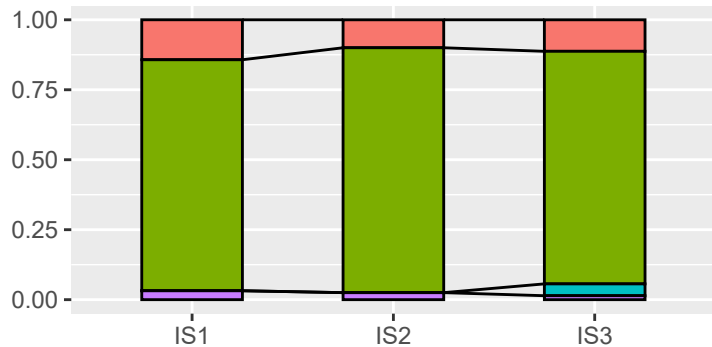

Stage Stage I Stage II Stage III Stage IV

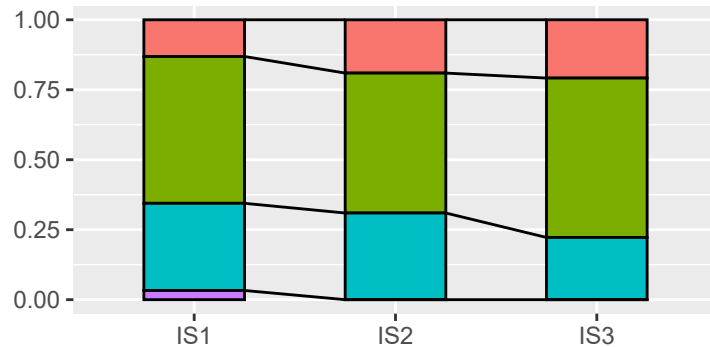

Grade G1 G2 G3 G4

Supplement: Supplementary Figure 1 — The distribution of the expression levels of four key genes in the innate immune regulatory cGAS-STING signaling pathway across three ISs. [file DataSheet_1.zip › Supplementary materials/FigS2.pdf]

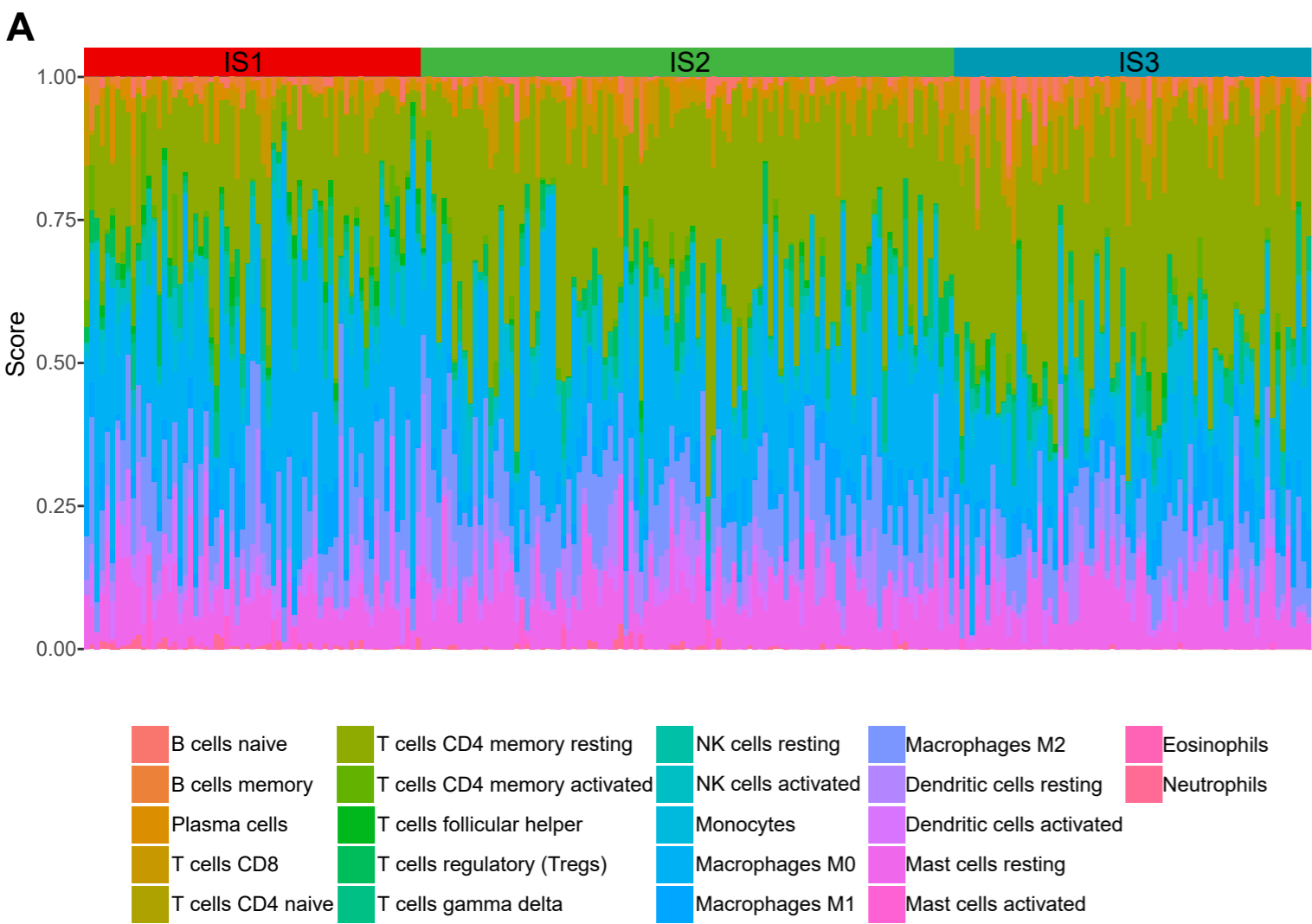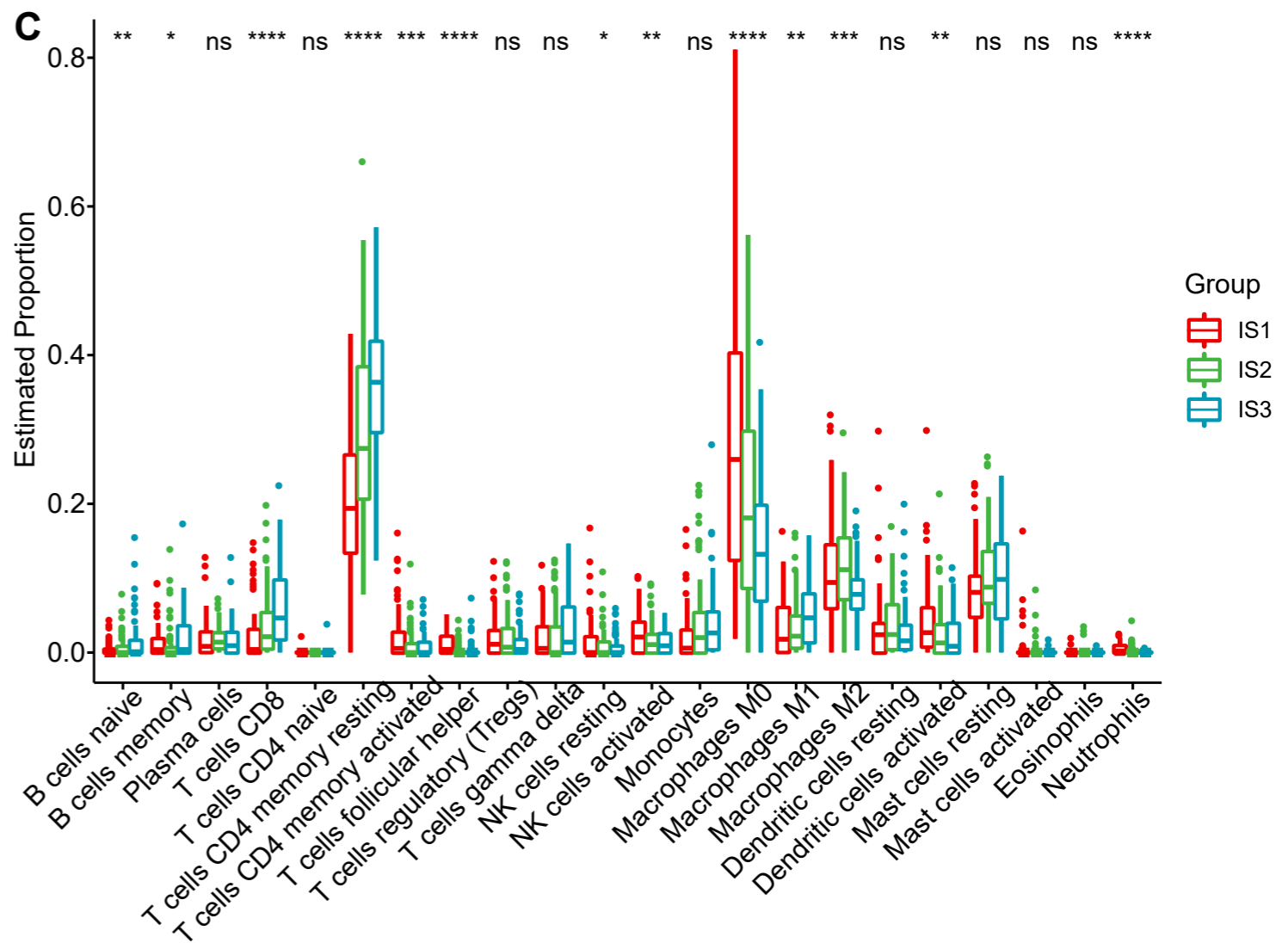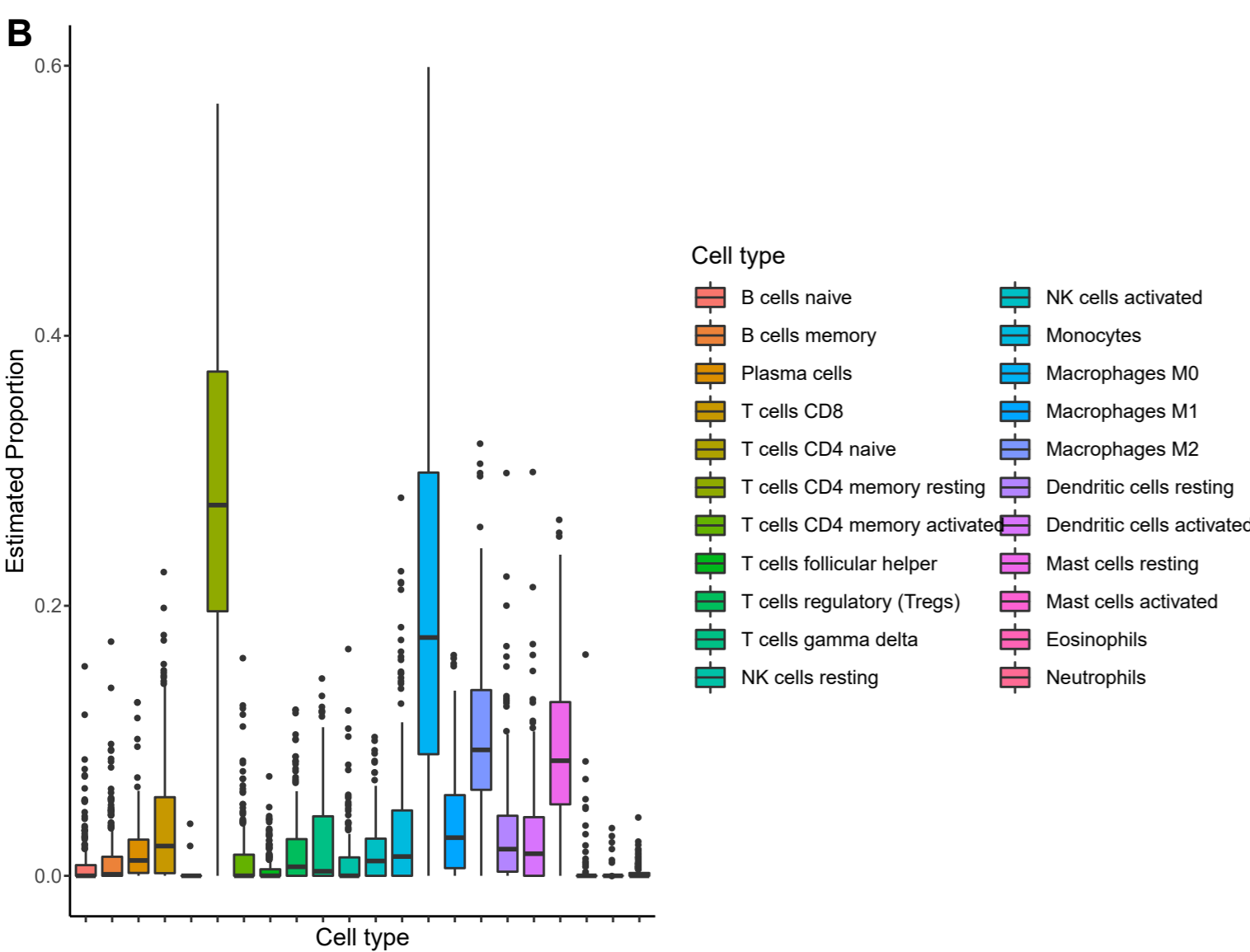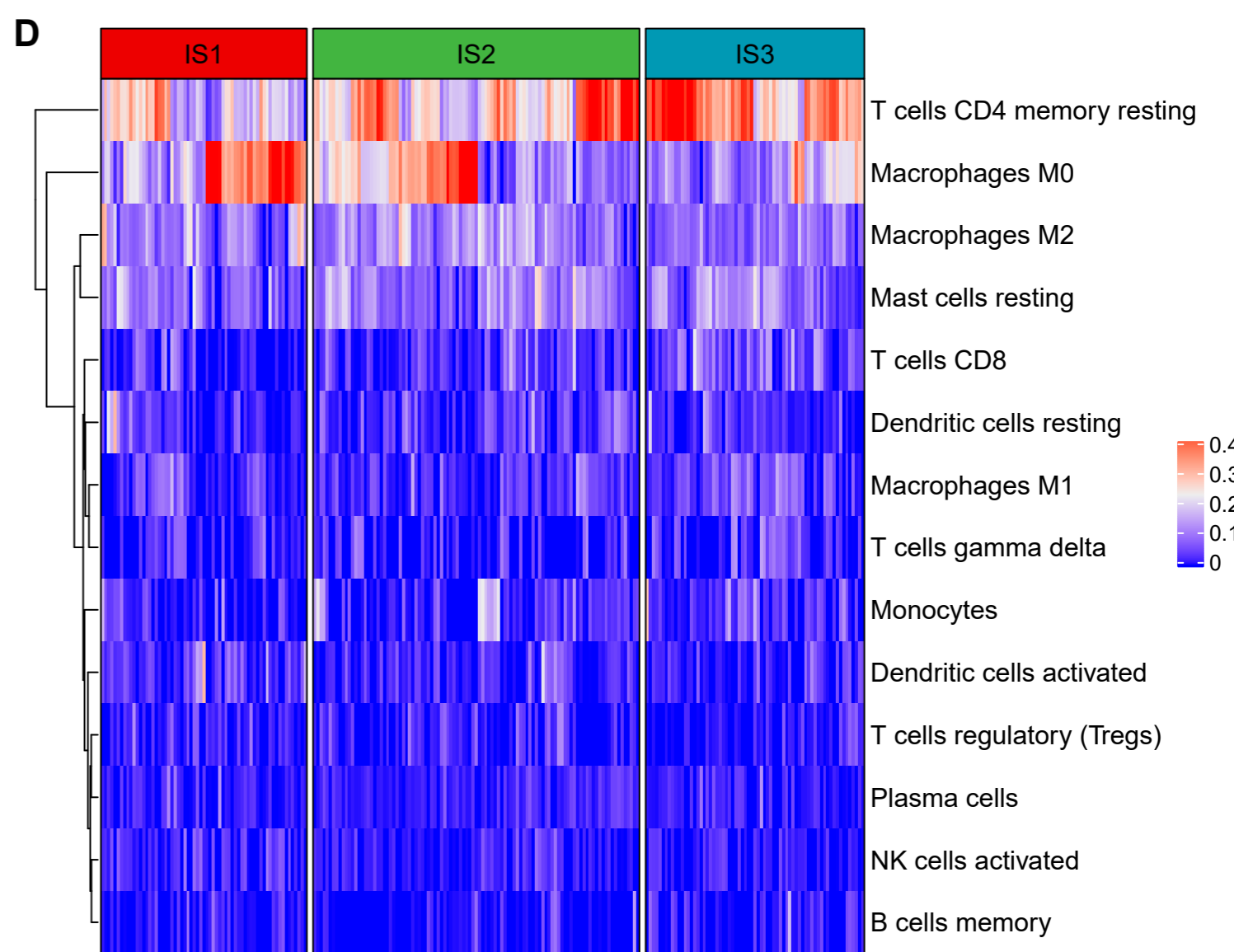

Supplement: Supplementary Figure 1 — The distribution of the expression levels of four key genes in the innate immune regulatory cGAS-STING signaling pathway across three ISs. [file DataSheet_1.zip › Supplementary materials/FigS3.pdf]

Group ■ IS1 ■ IS2 ■ IS3

**A** Kruskal-Wallis test  $p=0.3$

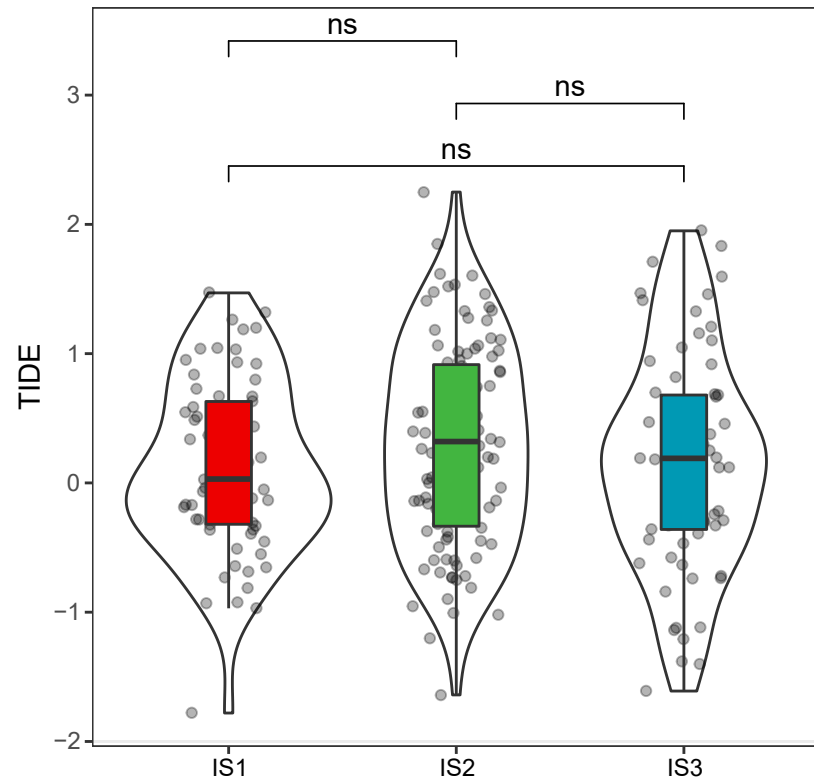

**B** Kruskal-Wallis test  $p=2.6e-17$

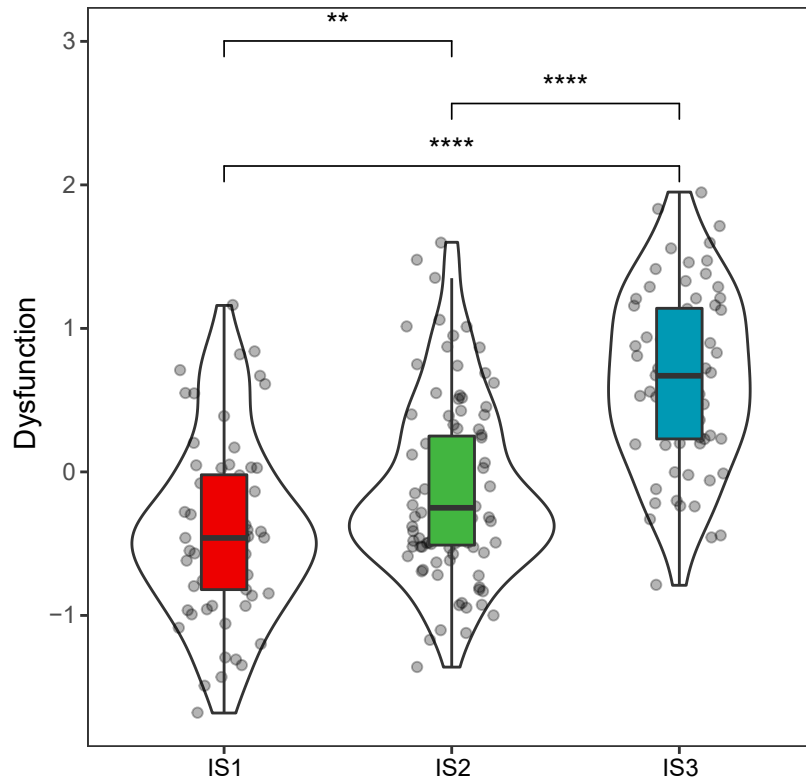

**C** Kruskal-Wallis test  $p=5e-07$

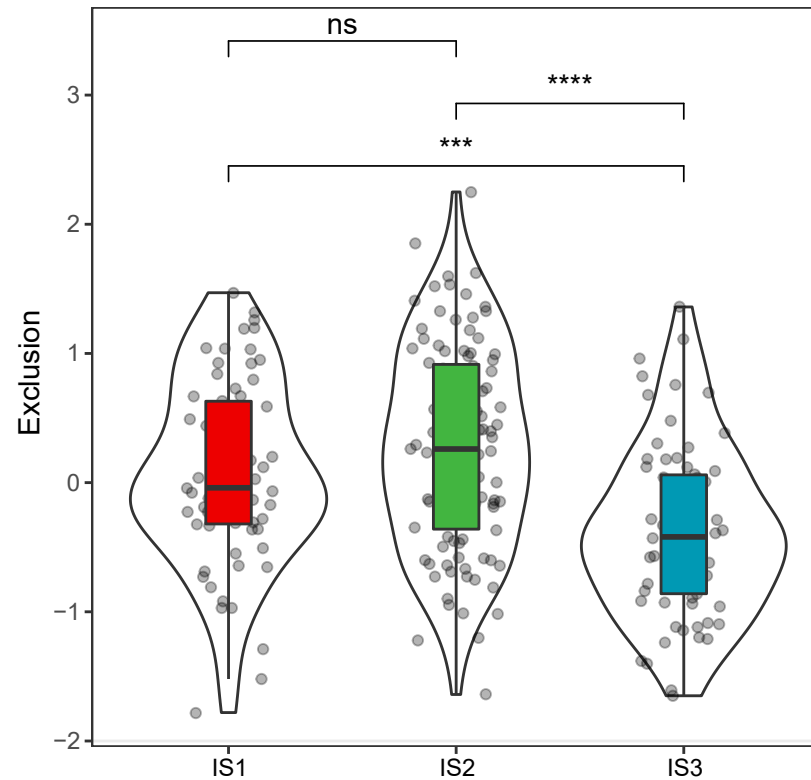

Supplement: Supplementary Figure 1 — The distribution of the expression levels of four key genes in the innate immune regulatory cGAS-STING signaling pathway across three ISs. [file DataSheet_1.zip › Supplementary materials/FigS4.pdf]

**A****Sample clustering to detect outliers**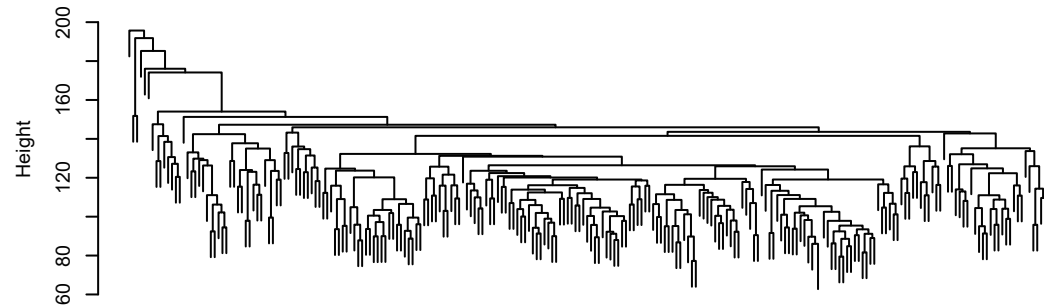**B****Cluster Dendrogram**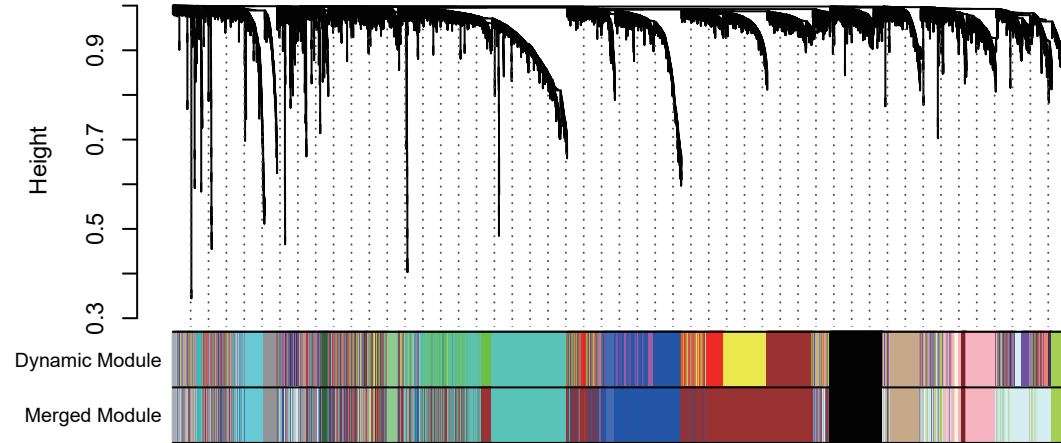

Supplement: Supplementary Figure 1 — The distribution of the expression levels of four key genes in the innate immune regulatory cGAS-STING signaling pathway across three ISs. [file DataSheet_1.zip › Supplementary materials/FigS7.pdf]

**A**

Top10 Biological Process

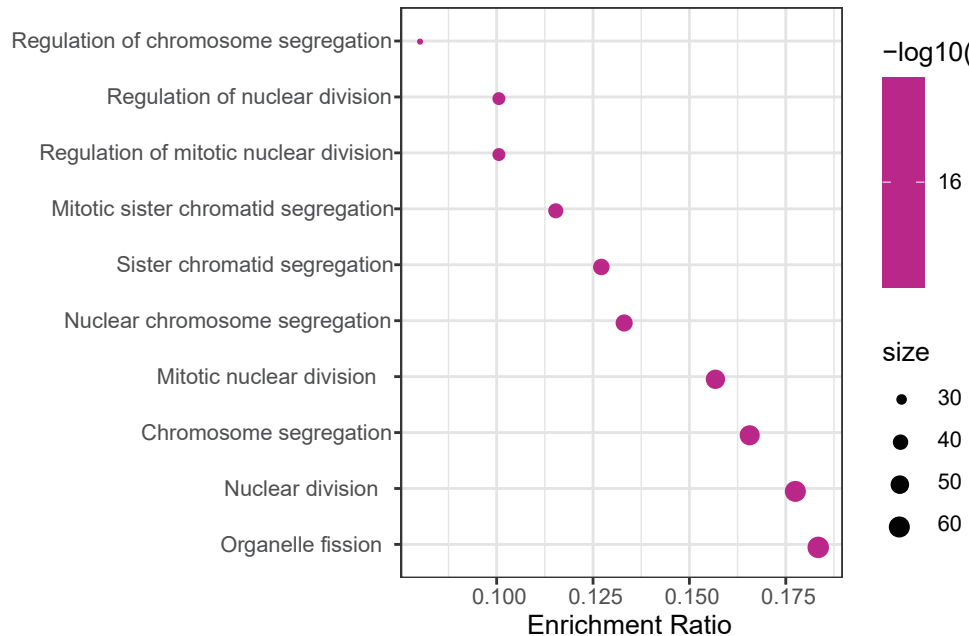**B**

Top 10 KEGG pathway

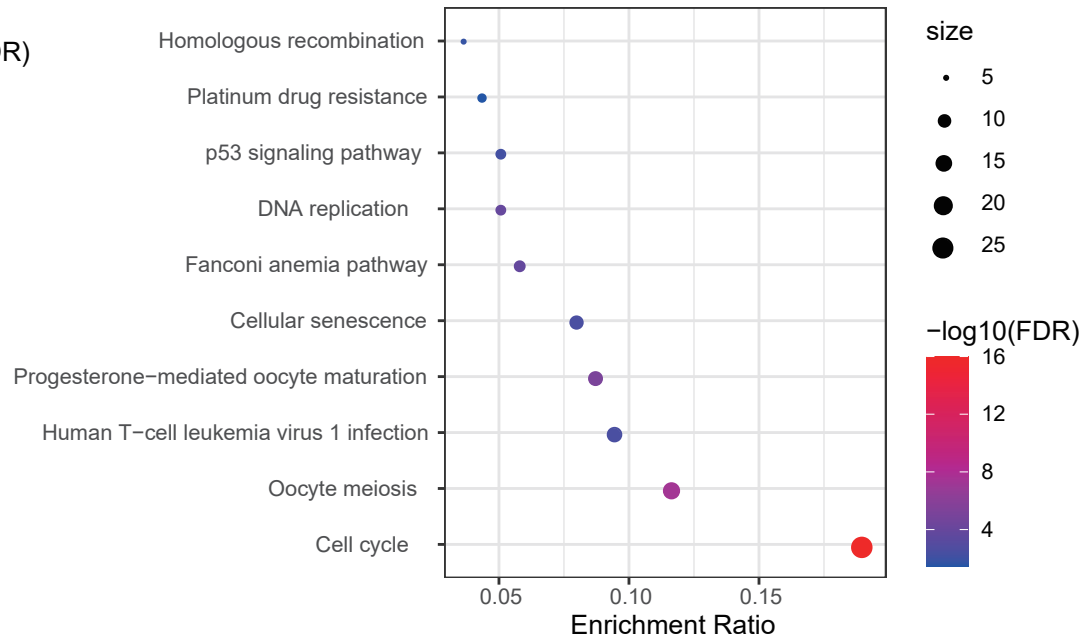

Supplement: Supplementary Figure 1 — The distribution of the expression levels of four key genes in the innate immune regulatory cGAS-STING signaling pathway across three ISs. [file DataSheet_1.zip › Supplementary materials/FigS8.pdf]

**CTBP1-DT**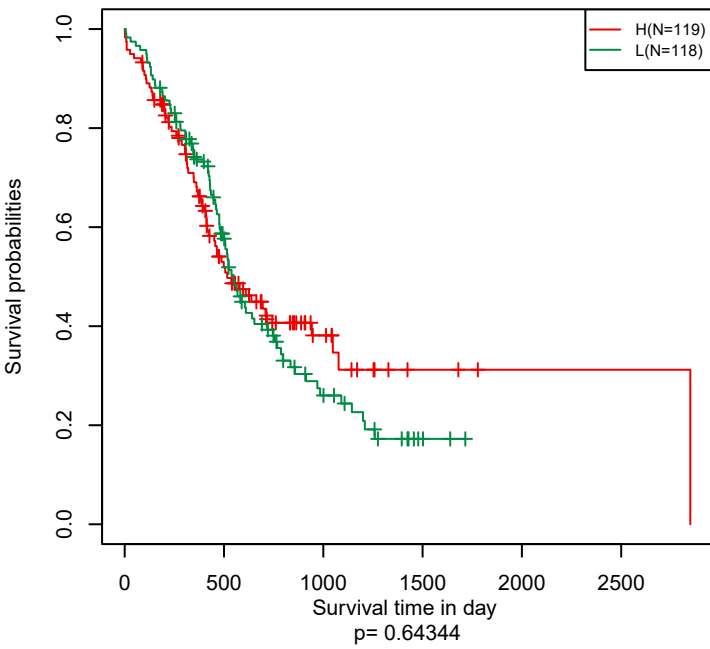**ZNF165**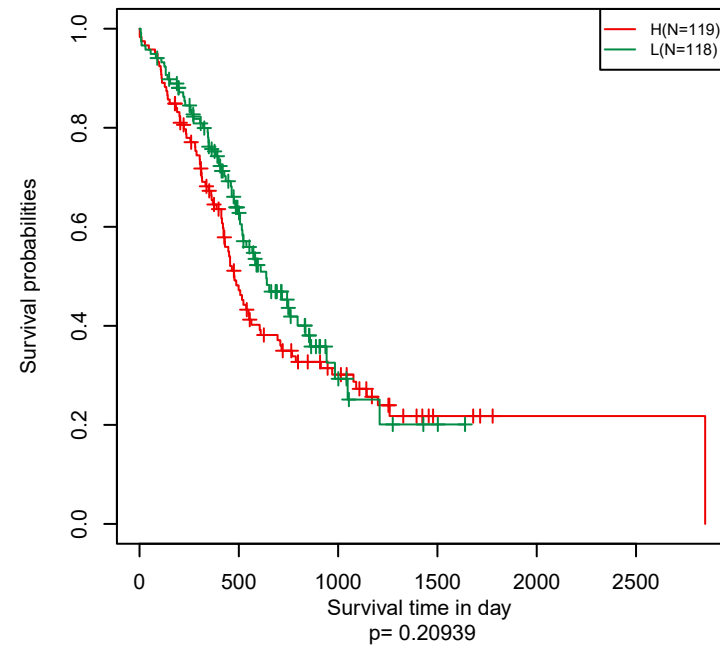**TPSB2**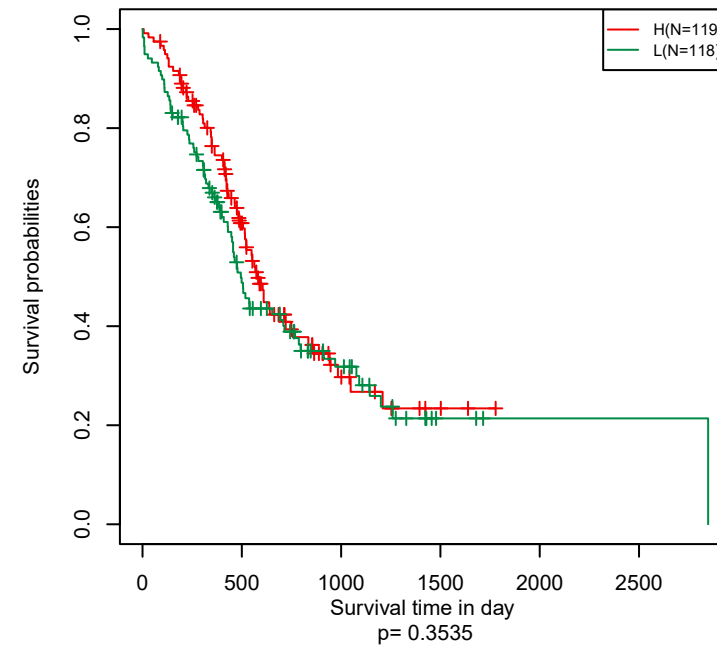**ZNF195**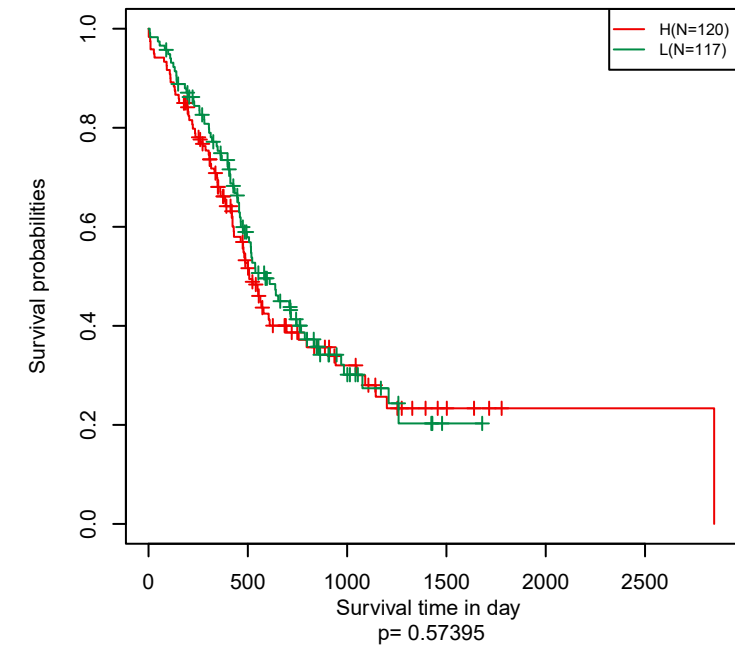**TRAF1**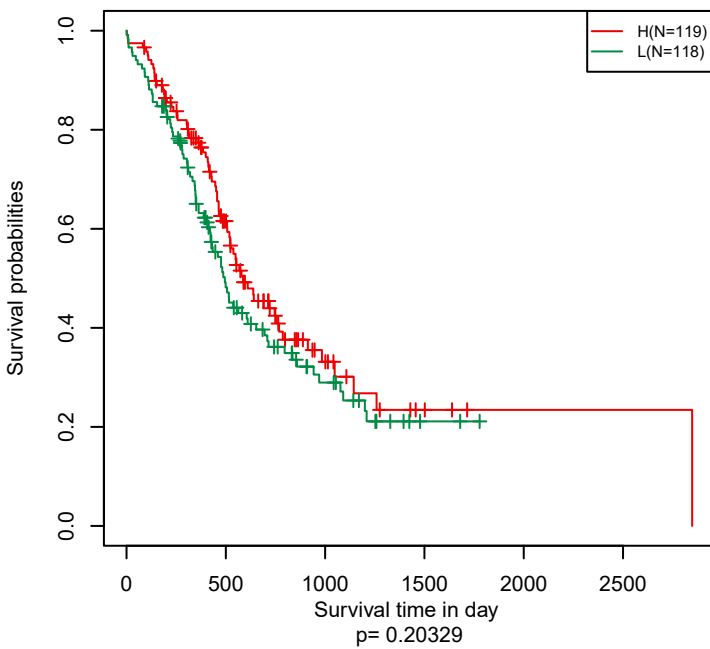**TRAF3IP3**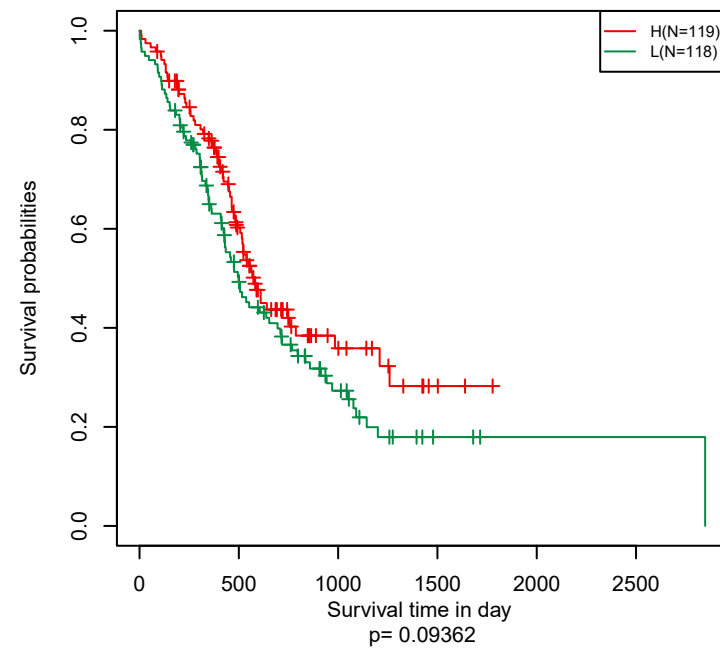**ZNF271P**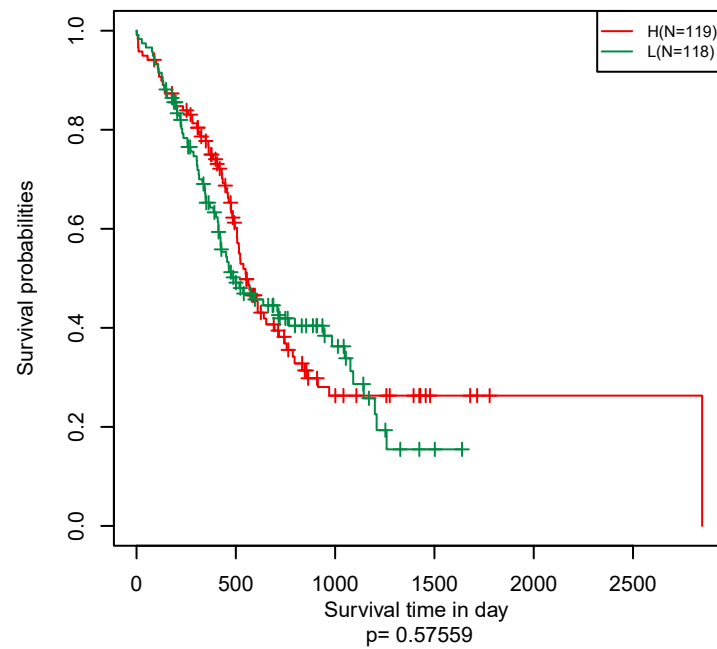

Supplement: Supplementary Figure 1 — The distribution of the expression levels of four key genes in the innate immune regulatory cGAS-STING signaling pathway across three ISs. [file DataSheet_1.zip › Supplementary materials/FigS9.pdf]
