# Supplementary material for: Immunological Classification of Pancreatic Carcinomas to Identify Immune Index and Provide a Strategy for Patient Stratification
Source: Front Immunol. 2022 Jan 17;12:719105. doi: 10.3389/fimmu.2021.719105 (PMC8801451; doi:10.3389/fimmu.2021.719105)
Supplement: Supplementary Figure 1 — The distribution of the expression levels of four key genes in the innate immune regulatory cGAS-STING signaling pathway across three ISs. [file FigS5.pdf › Supplementary materials/FigS5.pdf]

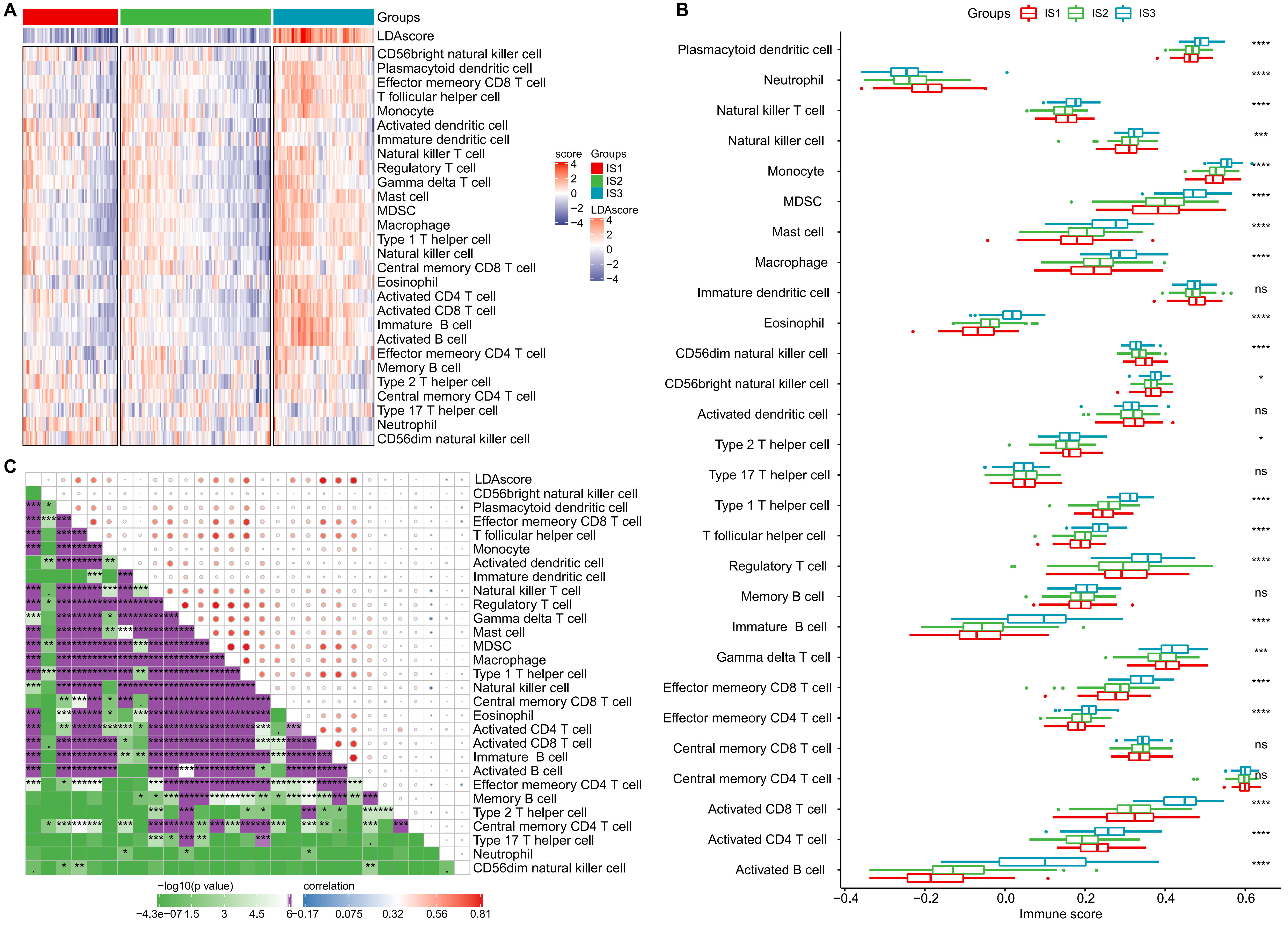
A
Groups
LDAscore
CD56bright natural killer cell
Plasmacytoid dendritic cell
Effector memeory CD8 T cell
T follicular helper cell
Monocyte
Activated dendritic cell
Immature dendritic cell
Natural killer T cell
Regulatory T cell
Gamma delta T cell
Mast cell
MDSC
Macrophage
Type 1 T helper cell
Natural killer cell
Central memory CD8 T cell
Eosinophil
Activated CD4 T cell
Activated CD8 T cell
Immature B cell
Activated B cell
Effector memeory CD4 T cell
Memory B cell
Type 2 T helper cell
Central memory CD4 T cell
Type 17 T helper cell
Neutrophil
CD56dim natural killer cell
score
4
2
0
−2
−4
Groups
IS1
IS2
IS3
LDAscore
4
2
0
−2
−4
B
Groups
IS1
IS2
IS3
Plasmacytoid dendritic cell ****
Neutrophil ****
Natural killer T cell ****
Natural killer cell ***
Monocyte ****
MDSC ****
Mast cell ****
Macrophage ****
Immature dendritic cell ns
Eosinophil ****
CD56dim natural killer cell ****
CD56bright natural killer cell *
Activated dendritic cell ns
Type 2 T helper cell *
Type 17 T helper cell ns
Type 1 T helper cell ****
T follicular helper cell ****
Regulatory T cell ****
Memory B cell ns
Immature B cell ****
Gamma delta T cell ***
Effector memeory CD8 T cell ****
Effector memeory CD4 T cell ****
Central memory CD8 T cell ns
Central memory CD4 T cell ns
Activated CD8 T cell ****
Activated CD4 T cell ****
Activated B cell ****
−0.4
−0.2
0.0
0.2
0.4
0.6
Immune score
C
LDAscore
CD56bright natural killer cell
Plasmacytoid dendritic cell
Effector memeory CD8 T cell
T follicular helper cell
Monocyte
Activated dendritic cell
Immature dendritic cell
Natural killer T cell
Regulatory T cell
Gamma delta T cell
Mast cell
MDSC
Macrophage
Type 1 T helper cell
Natural killer cell
Central memory CD8 T cell
Eosinophil
Activated CD4 T cell
Activated CD8 T cell
Immature B cell
Activated B cell
Effector memeory CD4 T cell
Memory B cell
Type 2 T helper cell
Central memory CD4 T cell
Type 17 T helper cell
Neutrophil
CD56dim natural killer cell
−log10(p value)
−4.3e−07
1.5
3
4.5
6
correlation
−0.17
0.075
0.32
0.56
0.81
