# Supplementary material for: Immunological Classification of Pancreatic Carcinomas to Identify Immune Index and Provide a Strategy for Patient Stratification
Source: Front Immunol. 2022 Jan 17;12:719105. doi: 10.3389/fimmu.2021.719105 (PMC8801451; doi:10.3389/fimmu.2021.719105)
Supplement: Supplementary Figure 1 — The distribution of the expression levels of four key genes in the innate immune regulatory cGAS-STING signaling pathway across three ISs. [file FigS6.pdf › Supplementary materials/FigS6.pdf]

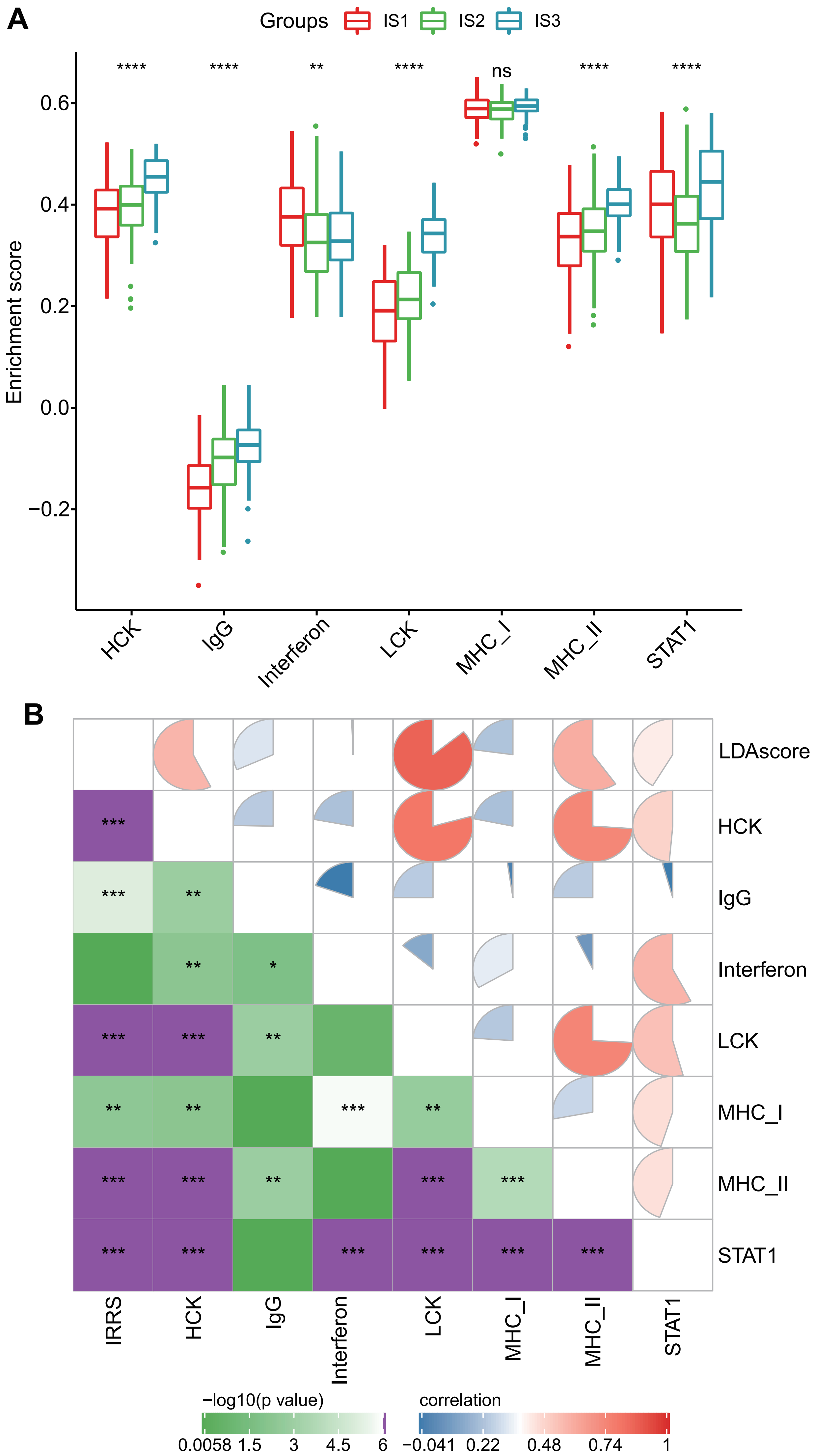
A
Groups
IS1
IS2
IS3
Enrichment score
0.6
0.4
0.2
0.0
−0.2
****
****
**
****
ns
****
****
HCK
IgG
Interferon
LCK
MHC_I
MHC_II
STAT1
B
LDAscore
HCK
IgG
Interferon
LCK
MHC_I
MHC_II
STAT1
IRRS
HCK
IgG
Interferon
LCK
MHC_I
MHC_II
STAT1
−log10(p value)
0.0058 1.5 3 4.5 6
correlation
−0.041 0.22 0.48 0.74 1
